# Building inorganic supramolecular architectures using principles adopted from the organic solid state

**DOI:** 10.1107/S2052252517015494

**Published:** 2018-01-01

**Authors:** Marijana Đaković, Željka Soldin, Boris-Marko Kukovec, Ivan Kodrin, Christer B. Aakeröy, Nea Baus, Tamara Rinkovec

**Affiliations:** a Department of Chemistry, Faculty of Science, University of Zagreb, Horvatovac 102a, Zagreb, HR-10000, Croatia; b Department of Chemistry, Kansas State University, Manhattan, KS 66506, USA

**Keywords:** crystal engineering, hydrogen bonds, electrostatic potential, synthons, coordination compounds

## Abstract

In order to develop transferable and practical avenues for the assembly of coordination complexes into architectures with specific dimensionality, a strategy utilizing ligands capable of simultaneous metal coordination and self-complementary hydrogen bonding is presented. The three ligands used, 2(1*H*)-pyrazinone, 4(3*H*)-pyrimidinone and 4(3*H*)-quinazolinone, consistently deliver the required synthetic vectors in a series of Cd^II^ coordination polymers, allowing for reproducible supramolecular synthesis that is insensitive to the different steric and geometric demands from potentially disruptive counterions. In all nine crystallographically characterized compounds presented here, directional intermolecular N—H⋯O hydrogen bonds between ligands on adjacent complex building blocks drive the assembly and orientation of discrete building blocks into largely predictable topologies. Furthermore, whether the solids are prepared from solution or through liquid-assisted grinding, the structural outcome is the same, thus emphasizing the robustness of the synthetic protocol. The details of the molecular recognition events that take place in this series of compounds have been clearly delineated and rationalized in the context of calculated molecular electrostatic potential surfaces.

## Abstract 2

The possibility of converting crystal engineering principles that originate in the organic solid state into productive strategies for the effective supramolecular assembly of coordination complexes has been established through a range of complementary techniques. Calculated molecular electrostatic potentials, in conjunction with a systematic structural study, demonstrate that the existence and structural importance of the key hydrogen-bond interactions are not disrupted by the presence of metal cations and charge-balancing anions.

## 1. Introduction   

One of the primary challenges standing in the way of more predictable supramolecular assembly of metal-containing structures with precise metrics and topologies is the lack of transferable and robust guidelines for pre-planned synthesis based on non-covalent interactions (Brammer, 2004 ▸; Hosseini, 2005 ▸; Aakeröy, Chopade & Desper, 2013 ▸). In order to make us less reliant on serendipity when targeting a desired supramolecular architecture, we need to map out carefully the structural landscape that describes how different molecules recognize, interact and communicate (in a structural sense), even in the presence of the potentially disruptive counter-ions that regularly appear in metal–organic systems (Aakeröy *et al.*, 2009 ▸; Đaković *et al.*, 2011 ▸).

A relatively common approach to crystal engineering comprising coordination complexes employs ligands that are capable of forming reliable coordination–covalent bonds with metal ions, and which at the same time can participate in self-complementary non-covalent interactions (typically hydrogen bonds) that promote the directed assembly of discrete complex ions into one, two or three-dimensional architectures (Brammer *et al.*, 2002 ▸; Kukovec *et al.*, 2016 ▸). Although such strategies are relatively straightforward and logical, and the targets are seemingly attainable and realistic, the reversibility of non-covalent interactions often conspires to produce outcomes that are neither desirable nor expected (Đaković *et al.*, 2013 ▸).

The use of functional groups capable of self-complementary non-covalent interactions for propagating the assembly of discrete building blocks into extended architectures is motivated by the fact that some chemical functionalities can be relied upon to be structurally consistent (Sarma & Desiraju, 2002 ▸). The extent to which a particular functional group is likely to form a specific synthon (Desiraju, 1995 ▸) can be elucidated through careful analysis of the Cambridge Structural Database (CSD) (Groom *et al.*, 2016 ▸; Aakeröy, Epa *et al.*, 2013 ▸). In this way, the hydrogen-bond preferences of numerous functional groups have been evaluated and quantified in order to develop supramolecular synthetic routes, especially in crystal engineering involving organic molecular solids (Shattock *et al.*, 2008 ▸; Aakeröy, Sinha *et al.*, 2013 ▸; Vishweshwar *et al.*, 2002 ▸).

**Figure 1:**
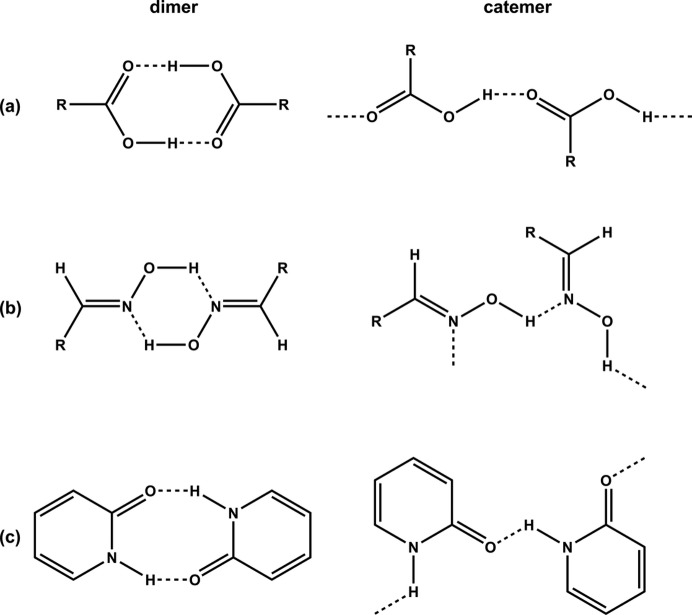
Hydrogen-bonded dimers and catemers of (*a*) carb­oxy­lic acids, (*b*) oximes and (*c*) 2(1*H*)-pyridinone.

If we want to maximize our chances of precisely controlling the main structural features of the product of a particular directed-assembly process, it is not sufficient simply to know how likely it is that a given functional group will form self-complementary interactions (Desiraju, 2001 ▸). In addition, since some functional groups are capable of forming geo­metrically different homomeric synthons, we also need to establish the relative propensities of these different structural options. For example, carb­oxy­lic acids and oximes prefer head-to-head 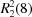 and 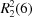 motifs, respectively, but both groups are also capable of producing one-dimensional catemeric chains, *C*(4) and *C*(3), respectively (Figs. 1 ▸ *a* and 1 ▸ *b*) [For full details of graph-set analysis of hydrogen bonds, see Bernstein *et al.* (1995 ▸)].

Synthon polymorphism (Sreekanth *et al.*, 2007 ▸; Gryl *et al.*, 2008 ▸; Mukherjee & Desiraju, 2011 ▸) can have dramatic structural effects on the exact nature of the overall resulting assembly, which in turn can produce solid forms with unwanted or sub-optimum properties. It is therefore very important, from a supramolecular synthetic perspective, to know (i) what the relative frequency of occurrence is for each option and (ii) what the resulting structural consequences are for each synthon type, because the properties of the bulk are directly connected to the structural consequences of the synthons. Conventional polymorphism highlights in similar ways the critical connection between structure and function and it is well known that manufacturers of high-value organic solid chemicals, such as the pharmaceutical industry, spend enormous resources on making sure that the solid-form landscape for each target is completely mapped out.

**Figure 2:**
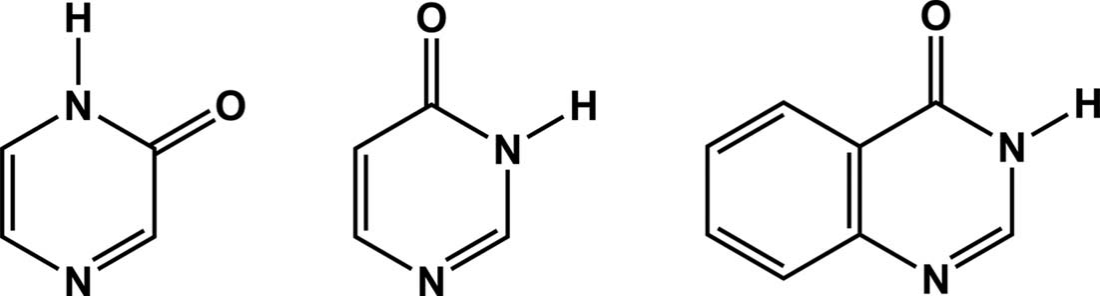
(Left) 2(1*H*)-pyrazinone, **2-pyz**, (middle) 4(3*H*)-pyrimidinone, **4-pym**, and (right) 4(3*H*)-quinazolinone, **4-quz**.

In this study, we focus our attention on three molecules that present a carbonyl group adjacent to a heterocyclic nitro­gen atom, thereby creating a functionality that can engage in either head-to-head dimers or catemeric chains (Fig. 1 ▸ *c*). The three tectons, 2(1*H*)-pyrazinone (**2-pyz**), 4(3*H*)-pyrimidinone (**4-pym**) and 4(3*H*)-quinazolinone (**4-quz**) (Fig. 2 ▸), are all multi-functional in the sense that they can act as ligands in transition metal complexes while delivering vectors for supramolecular assembly through hydrogen-bond based synthons.

**Figure 3:**
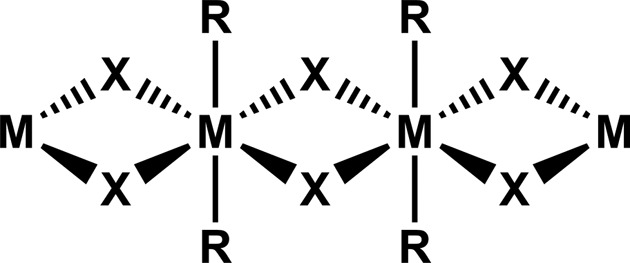
The targeted one-dimensional Cd^II^-based coordination polymer (*M* = Cd, *X* = halide ion and *R* = **2-pyz**, **4-pym** or **4-quz**.

In order to make reliable inferences from molecular structure to crystal structure it is necessary to minimize the potential for compositional and structural diversity that is often present in metal-containing systems. Therefore, we incorporated a structurally reliable invariant that would act as a robust foundation from which we could directly examine the non-covalent aspects (synthon preferences) of these pyridinone-type ligands. We opted to build the central structural invariant around a Cd^II^ halide framework as this would present a reasonably predictable one-dimensional coordination polymer (Englert, 2010 ▸) (Fig. 3 ▸).

**Figure 4:**
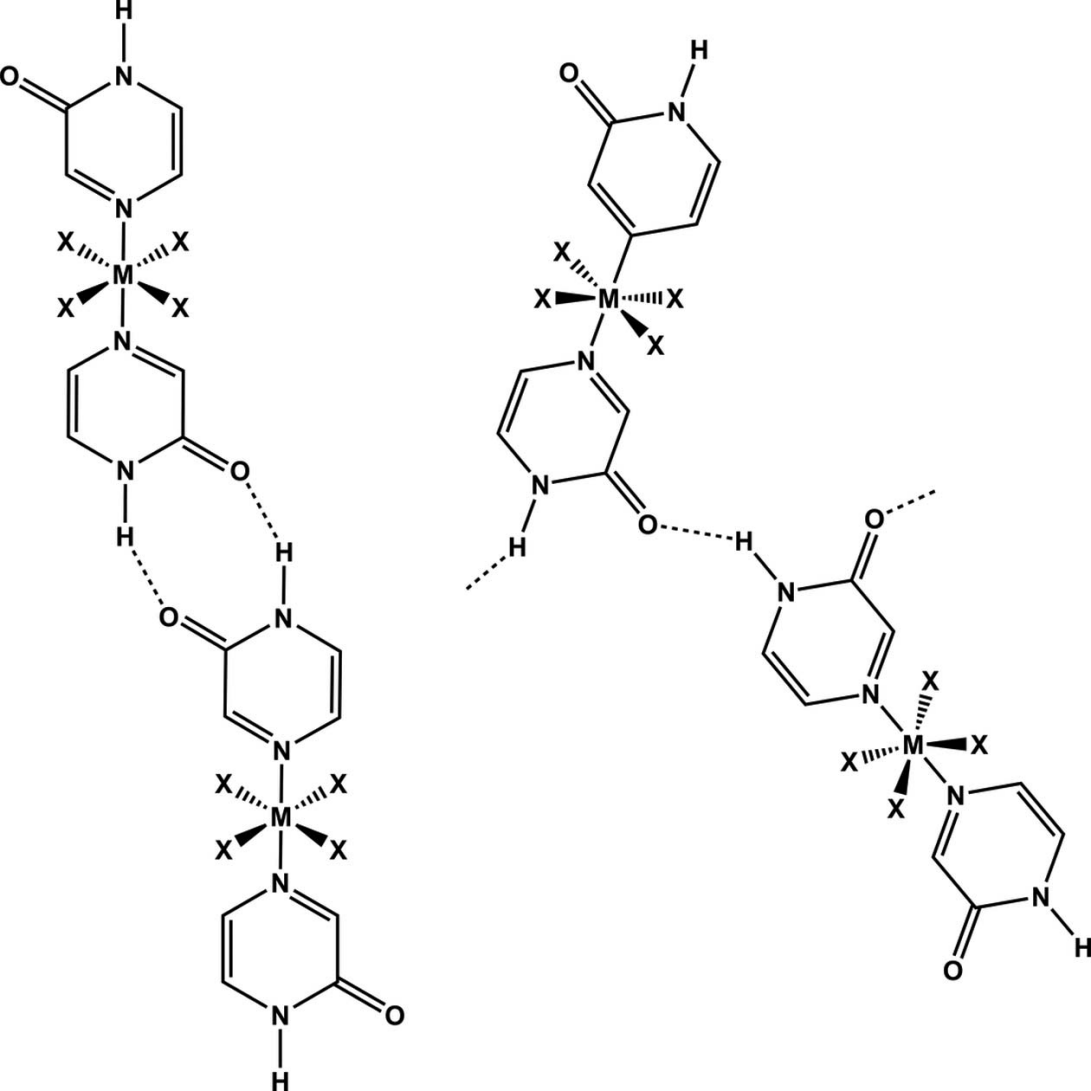
The two synthon types, (left) the 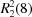 dimer and (right) the *C*(4) catemer, formed between two basic metal-based building units equipped with pyrazinone ligands. The pyrimidinone and quinazolinone ligands are prone to exhibit the same hydrogen-bond motifs.

In addition, the octahedral coordination requirement of Cd^II^ cations offers the necessary anchoring points for two auxiliary ligands in the axial positions (Fig. 4 ▸). The supra­molecular chemistry of these ligands can then be examined in detail with a view to developing new strategies for reliable and versatile programmed assembly of metal-containing solid-state architectures.

The goals of this study are to control the details of the coordination chemistry around each metal cation and then to deliver a coordination network of consistent dimensionality and topology through complementary hydrogen bonds. We also want to determine if a relatively simple electrostatic view of the hydrogen bond will allow us to rationalize the observed interactions in metal-containing systems with a number of different (and potentially disruptive) hydrogen-bond acceptor sites.

## 2. Experimental   

### 2.1. Materials and methods   

All metal salts, precursors and solvents were purchased from commercial suppliers and used without further purification.

The cadmium(II) halide complexes with 2(1*H*)-pyrazinone, namely [CdCl_2_(2-pyz)_2_]*_n_* (**1**), [CdBr_2_(2-pyz)_2_]*_n_* (**2**) and [CdI_2_(2-pyz)_2_]*_n_* (**3**), with 4(3*H*)-pyrimidinone, namely [CdCl_2_(2-pym)_2_]*_n_* (**4**), [CdBr_2_(2-pym)_2_]*_n_* (**5**) and [CdI_2_(2-pym)_2_]*_n_* (**6**), and with 4(3*H*)-quinazolinone, namely [CdCl_2_(4-quz)_2_]*_n_* (**7**), [CdBr_2_(4-quz)_2_]*_n_* (**8**) and [CdI_2_(4-quz)_2_]*_n_* (**9**), were prepared by two synthetic methods, liquid diffusion and solvent-assisted grinding.

**Liquid diffusion synthesis**. An aqueous solution of the cadmium(II) salt (1 ml) was layered with an ethanol solution of the ligand (2 ml). Solutions were further layered with acetone (1.5 ml) and left standing at room temperature. X-ray quality crystals were harvested after a period of 7–15 d.

**Mechanochemical synthesis**. Cadmium(II) salts and ligands in an equimolar ratio were ground in a stainless steel jar (10 ml in volume; using two stainless steel grinding balls of 7 mm in diameter) with the assistance of 100 µl of ethanol. A Retch MM200 grinder operating at 25 Hz frequency was used for the grinding experiments.

Full details of the syntheses can be found in the supporting information.

### 2.2. X-ray structure analysis   

Single crystals were mounted in a random orientation on a glass fibre using Paratone oil. Data collections were carried out on an Oxford Diffraction Xcalibur four-circle kappa geometry single-crystal diffractometer with a Sapphire 3 CCD detector using graphite monochromated Mo *K*α (λ = 0.71073 Å) radiation and applying the *CrysAlisPro* Software system (Agilent Technologies Ltd, 2014 ▸; Rigaku Oxford Diffraction Ltd, 2015 ▸), at 296 (2) K for compounds **2**, **3**, **5** and **9**, 200 (2) K for **1** and **6**, or 150 (2) K for **8**. Data reduction, including absorption correction, was done using *CrysAlisPRO*. The structures were solved with *SHELXS-2014* (Sheldrick, 2015 ▸) and refined on *F* ^2^ using *SHELXL-2014*. Table S1 in the supporting information reports all the crystal data, details of the data collection, and a full summary of the intensity data collection and structure refinement for the crystal structures of **1**–**3**, **5**, **6**, **8** and **9**. Displacement ellipsoid plots were drawn at the 50% probability level (*Mercury 3.3*; Macrae *et al.*, 2008 ▸). CCDC Nos. 1559137–1559143 contain the supplementary crystallographic data. These data can be obtained free of charge from the Cambridge Crystallographic Data Centre *via* http://www.ccdc.cam.ac.uk/data_request/cif.

### 2.3. Computational details   

The geometry of the coordination polymer unit was optimized by employing a one-dimensional periodic boundary condition (PBC) in *GAUSSIAN09* (Frisch *et al.*, 2009 ▸). The Perdew–Burke–Ernzerhof exchange and correlation functional (PBE) (Perdew *et al.*, 1996 ▸, 1997 ▸) was used, together with the Stuttgart/Dresden effective core potential basis set (SDD). After optimization, the one-dimensional coordination polymer was modelled as a unit with seven octahedrally coordinated cadmium centres to obtain more accurate mol­ecular electrostatic potential (MEP) values. Two monocationic metals (like Na^+^) were placed on each of the two ends, ensuring zero total charge of the molecule. With an increasing number of side molecules, the MEP values of the central unit atoms converged to reported values and the effect of the infinite chain termination almost vanished. More details about this will be published in a forthcoming paper. The MEP maps were visualized in *GaussView 5.0* (Dennington *et al.*, 2009 ▸). The MEP at a specific point on the 0.002 a.u. isodensity surface is given by the electrostatic potential energy (in kJ mol^−1^) that a positive unit charge would experience at that point. A continuous colour spectrum is used to assign different values of electrostatic potential energy values. The most negative values are coloured red and the most positive values are coloured blue.

The geometries of tetramers and molecular pairs were extracted from experimental crystallographic data after normalization of bond lengths with hydrogen to values obtained from neutron diffraction experiments. Single-point calculations and optimizations of ligands were performed in *GAUSSIAN09* using the M06-2X (Zhao & Truhlar, 2008 ▸) functional with the def2-TZVP basis set (Feller, 1996 ▸; Schuchardt *et al.*, 2007 ▸). A quantum theory of atoms in molecules (QTAIM) analysis (Bader, 1990 ▸) was performed with the *AIMAll* programme (Todd, 2016 ▸).

## 3. Results   

### 3.1. Structural studies   

**Figure 5:**
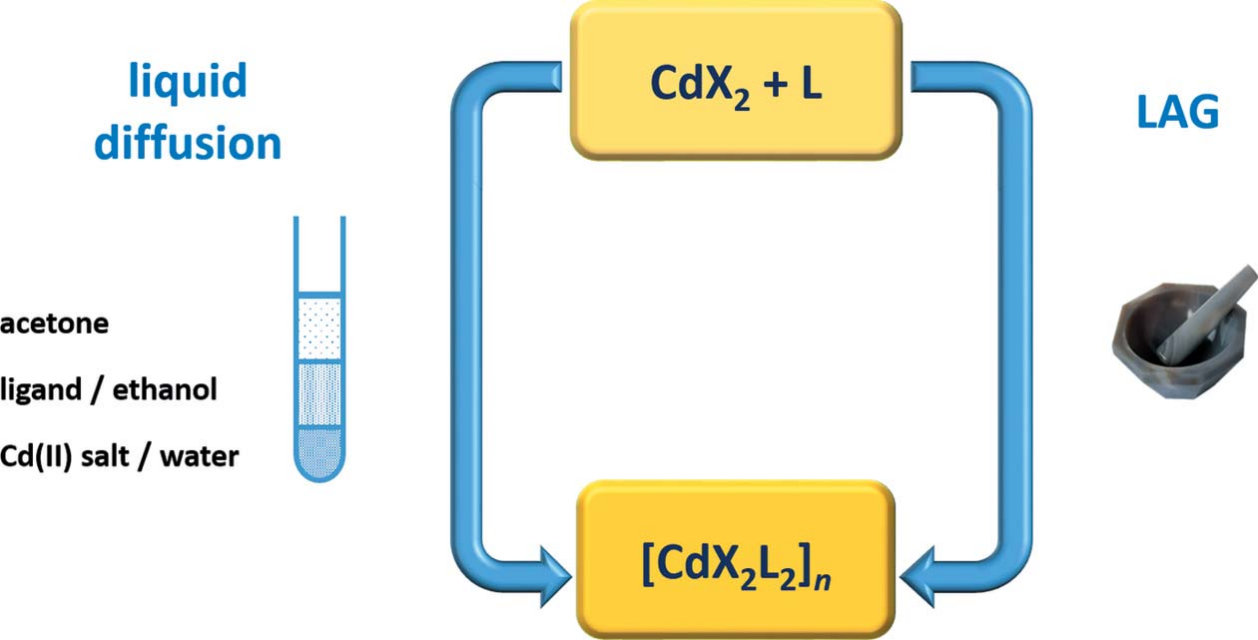
The synthetic pathways for complexes **1**–**9**. Both methods yield the exact same products, a polymeric [Cd*X*_2_ *L*_2_]*_n_* type of product (*X* = Cl, Br, I; *L* = 2-pyz, 4-pym, 4-quz) for **1**–**8**, and a monomeric complex [CdI_2_(4-quz)_2_] for **9**.

A series of cadmium(II) halide complexes with 2(1*H*)-pyrazinone (**1**–**3**), 4(3*H*)-pyrimidinone (**4**–**6**) and 4(3*H*)-quinazolinone (**7**–**9**) was prepared *via* two synthetic routes, (i) a liquid diffusion synthesis from a water–ethanol–acetone mixture, and (ii) ethanol-assisted grinding of a mixture of the starting compounds [*i.e.* cadmium(II) salts and organic ligands] in an equimolar ratio (Fig. 5 ▸) (Braga & Grepioni, 2005 ▸; Friščić, 2010 ▸). Both routes resulted in the same complexes in all nine cases **1**–**9**, emphasizing the highly robust and effective nature of the synthetic protocol. To avoid any potential solvent–solute bias, the same layering procedure was used for all the liquid diffusion experiments (2:4:3 water–ethanol–acetone layers). X-ray quality single crystals were harvested for all complexes (apart from **4**) from the diffusion experiments after a period of 1–2 weeks.

**Figure 6:**
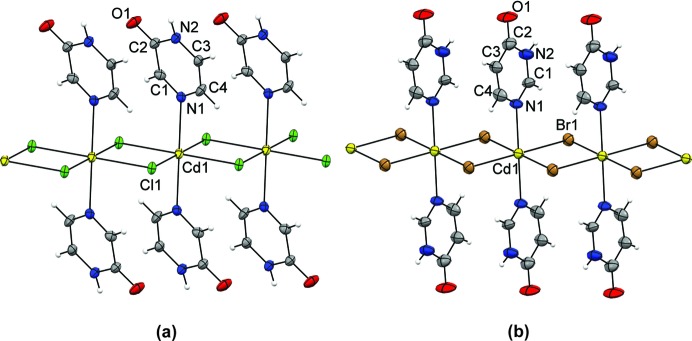
Metal-containing building units (*ORTEP*-style plots obtained using *Mercury 3.3*) equipped with pyrazinone (pyz) and pyrimidinone (pym) ligands, (*a*) [CdCl_2_(2-pyz)_2_]*_n_* (**1**) and (*b*) [CdBr_2_(4-pym)_2_]*_n_* (**5**), respectively. The crystal structures of [CdBr_2_(2-pyz)_2_]*_n_* (**2**) and [CdI_2_(2-pyz)_2_]*_n_* (**3**) are isostructural with each other and with **1**, and the structure of [CdI_2_(4-pym)_2_]*_n_* (**6**) is near-identical to that of **5**.

The structure determinations confirmed the expected one-dimensional polymeric chains as primary building units, with the cadmium(II) cations being octahedrally coordinated to four bridging halide ions and two *trans*-oriented organic ligands (Fig. 6 ▸). The ligands are in both cases coordinated *via* the nitro­gen atom distant to the carbonyl oxygen atom, *i.e.* the nitro­gen atom *meta* to the pyrazinone (**1**–**3**) and *para* to the pyrimidinone (**5**–**6**) oxygen atom. The only structural variances within **1**–**3** and **5**–**6** arise from the differences in the halide radii that affect the Cd^II^⋯Cd^II^ distances which, in turn, cause an increased ‘tilting’ of the aromatic rings upon moving from **1** to **3**, and from **5** to **6**.

**Figure 7:**
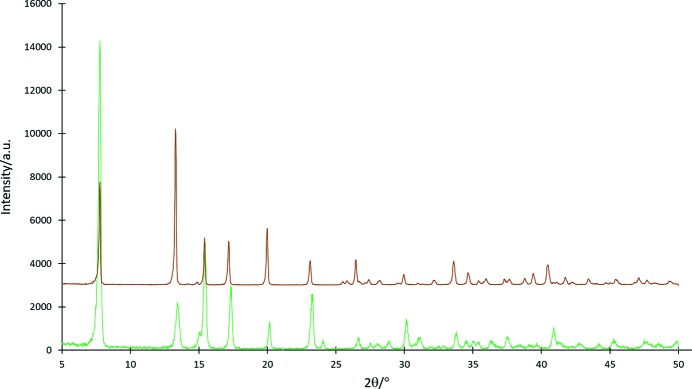
Overlay of the PXRD traces of [CdCl_2_(4-pym)_2_]*_n_* (**4**) (experimental; bottom) and [CdBr_2_(4-pym)_2_]*_n_* (**5**) (calculated; top).

Even though the crystal structure of **4** could not be obtained, a comparison of the powder X-ray diffraction (PXRD) traces of **4** and **5** (Fig. 7 ▸) strongly suggests that they are in fact isostructural.

**Figure 8:**
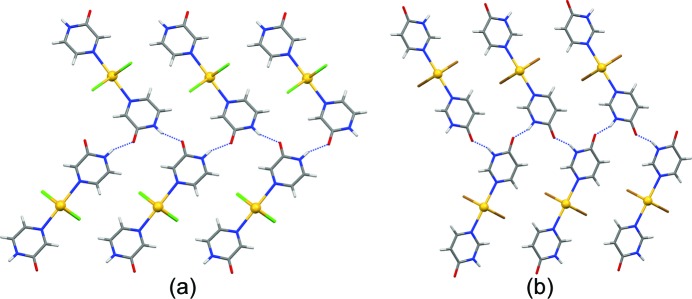
The relative orientations of adjacent polymeric chains (*Mercury 3.3*, capped sticks) in the crystal structures of (*a*) [CdCl_2_(2-pyz)_2_]*_n_* (**1**) and (*b*) [CdBr_2_(2-pym)_2_]*_n_* (**5**) linked *via* one single-point N—H⋯O hydrogen bond forming extended *C*(4) chains. [CdBr_2_(2-pyz)_2_]*_n_* (**2**) and [CdI_2_(2-pyz)_2_]*_n_* (**3**) exhibit the same supramolecular architecture as **1**, and [CdI_2_(2-pym)_2_]*_n_* (**6**) the same as **5**.

In all crystal structures **1**–**3** and **5**–**6**, the nearest neighbouring polymeric building units are linked *via* single-point N—H⋯O hydrogen bonds (Table 1 ▸) between adjacent pyrazinone or pyrimidinone groups, respectively, resulting in catemeric *C*(4) motifs (Fig. 8 ▸).

**Figure 9:**
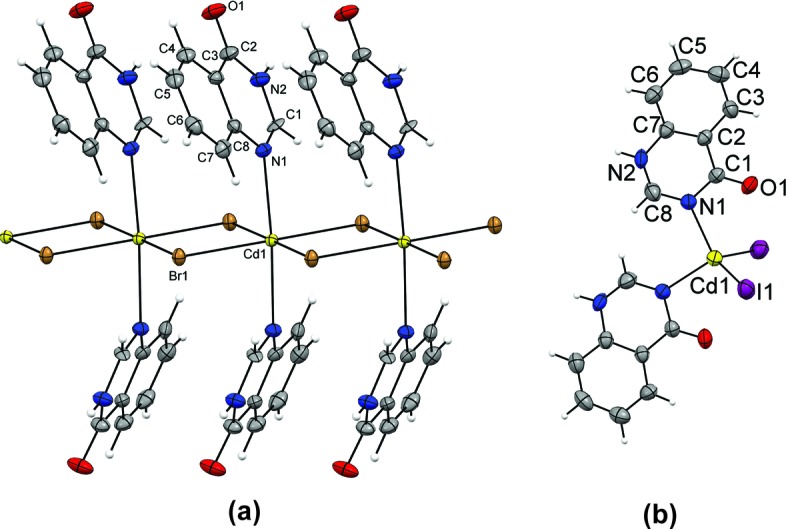
Metal-containing building units (*ORTEP*-style plots obtained using *Mercury 3.3*) bearing quinazolinone ligands. (*a*) [CdBr_2_(4-quz)_2_]*_n_* (**8**) and (*b*) [CdI_2_(4-quz)_2_] (**9**). The crystal structure of [CdCl_2_(4-quz)_2_]*_n_* (**7**) is isostructural with **8**.

Complex **8** displays essentially the same crystal structure as that previously reported for **7** (refcode NALFEN; Turgunov & Englert, 2010 ▸) and comprises targeted one-dimensional polymeric CdBr_2_ chains as core units with the expected octahedral geometry around the cadmium(II) cations, including a *trans*-arrangement of the two quinazolinone ligands (coordinated *via* the nitro­gen atom *para* to the quinazolinone oxygen atom) (Fig. 9 ▸ *a*).

Finally, the structure determination of **9** reveals an unusual monomeric metal-containing building unit with an unexpected tetrahedral arrangement of two quinazolinone and two iodide ligands (Fig. 9 ▸ *b*). Interestingly, the quinazolinone ligands, being coordinated *via* N1 in **9**, display a different coordination mode compared with that observed for **7** and **8**. It is the only complex in the series with metal coordination *via* the nitro­gen atom *ortho* to the carbonyl oxygen atom.

**Figure 10:**
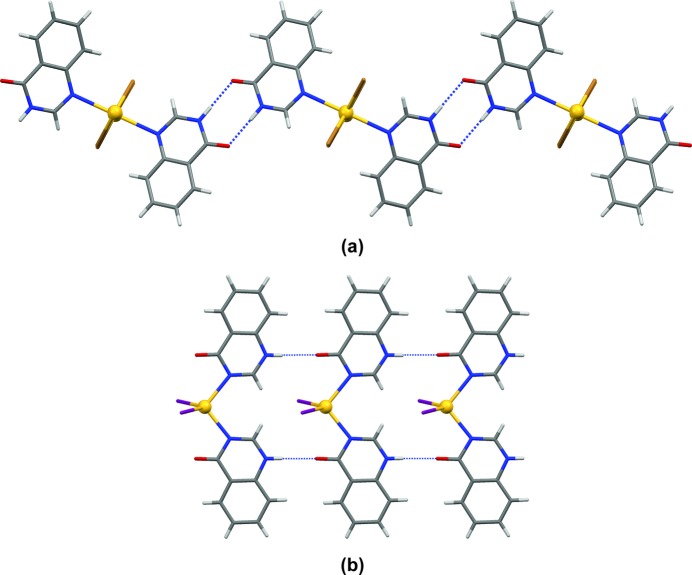
(*a*) Adjacent metal-containing building units (*Mercury 3.3*, capped sticks) in the crystal structure of [CdBr_2_(4-quz)_2_]*_n_* (**8**) linked by two N—H⋯O hydrogen bonds forming 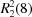 motifs. (*b*) Neighbouring metal-containing building units in the crystal structure of [Cd_2_I_2_(4-quz)_2_] (**9**) linked by two single-point N—H⋯O hydrogen bonds forming 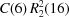 catemeric motifs.

Neighbouring polymeric units in **7** and **8** are linked *via* two hydrogen bonds of the N—H⋯O type forming head-to-head 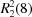 motifs (Fig. 10 ▸ *a*). Adjacent monomers of **9** are interconnected *via* the same supramolecular link, N—H⋯O hydrogen bonds, just forming somewhat different supra­molecular motifs in the form of extended chain-like 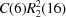 catemers (Fig. 10 ▸ *b*).

### 3.2. CSD survey   

**Figure 11:**
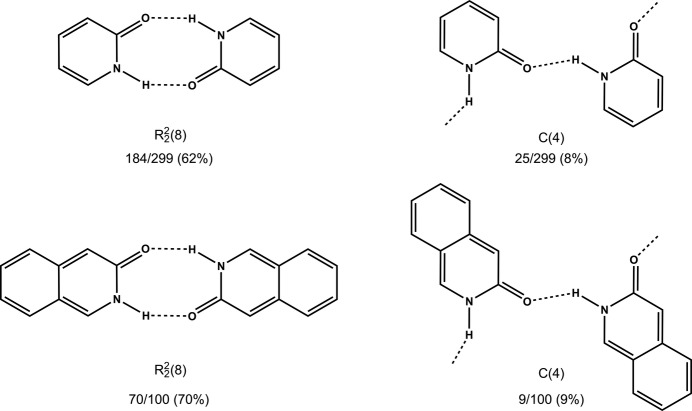
A survey of the CSD revealed a pronounced tendency for 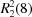 synthon formation for the pyridine- and quinoline-type fragments in purely organic solids.

To explore the extent to which it is possible to transfer hydrogen-bond synthons from organic to metal–organic systems, we retrieved information from the CSD (version 5.38, update November 2016; Groom *at al.*) on the propensity for hydrogen-bond motif formation of the three pyridinone-type ligands (**2-pyz**, **4-pym** and **4-quz**) in purely organic systems. The search was limited to pyridine- and quinoline-type fragments with the carbonyl group directly adjacent to a heterocyclic nitro­gen atom. The additional endocyclic nitro­gen atom in the heterocyclic rings (*para* to N in **2-pyz** and *meta* to N in **4-pym** and **4-quz**) is occupied through coordination to the metal centres. Therefore, as it would not have an impact on supramolecular motif formation, it was not included in the search fragments (Fig. 11 ▸).

The results showed that the ring motif 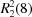 is more common than the *C*(4) catemer as it appears in 184 out of 299 times (62%) for the pyridine-type fragment, and in 70 out of 100 times (70%) for the quinoline analogue. The catemeric motif is present in only 25/299 (8%) and in 9/100 (9%) of the pyridine and quinoline fragments, respectively (Fig. 11 ▸).

### 3.3. Computational study   

To rationalize the supramolecular outcome of these reactions against a backdrop of MEP surfaces, while avoiding computationally expensive optimizations of the entirety of the crystal structure, we optimized the geometry of a one-dimensional coordination polymer unit by employing periodic boundary conditions. Then, a finite model of seven octa­hedrally coordinated cadmium centres with zero total charge was employed to obtain the more accurate MEP values. More details regarding the procedure are provided in the *Experimental* [Sec sec2] section.

**Figure 12:**
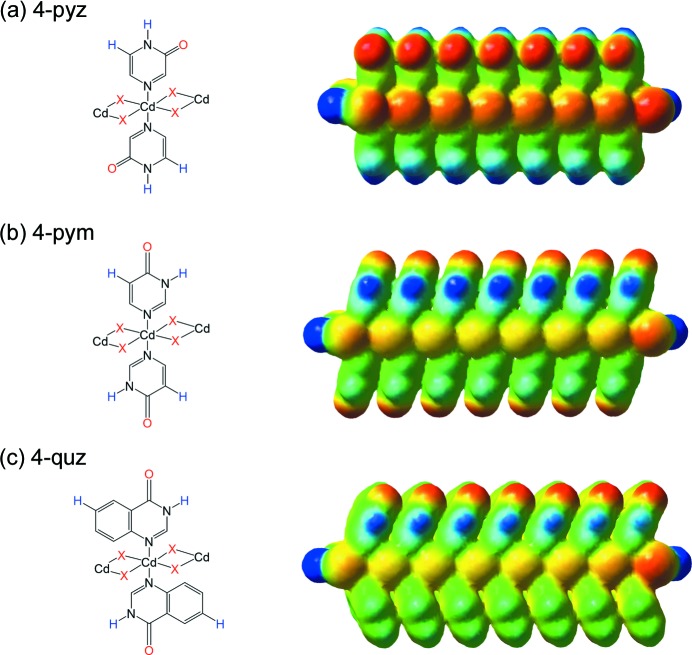
The positions of the atoms with the most positive (blue) and most negative (red) molecular electrostatic potential values for (*a*) pyrazinone- (in complexes **1**–**3**), (*b*) pyrimidinone- (in complexes **4**–**6**) and (*c*) quinazolinone-based units (in complexes **7** and **8**, and in the hypothetical model of complex **9**) presented for polymer fragments consisting of seven cadmium(II) centres for compounds **2**, **5** and **8**, respectively. Isodensity surface of 0.002 a.u., colour range −215 (red) to 310 kJ mol^−1^ (blue).

Fig. 12 ▸ shows the MEP maps for the polymeric fragments comprising seven cadmium(II) centres, mimicking an infinite coordination polymer chain. By employing three additional units placed on each side of the central cadmium(II) ion, the effect of deliberate termination of the polymeric chain is significantly reduced. The MEP values for the two potential acceptor sites, the coordinated halide anions and the carbonyl oxygen atoms, as well as the two most positive surface potentials for each compound are listed in Table 2 ▸. In all nine cases, the carbonyl oxygen atom carries the most negative potential and the halide anion the second most negative one, while the site of the most positive potential is always the hydrogen atom attached to an endocyclic nitro­gen atom. The second most positive potential site is located on an aromatic hydrogen atom, the position of which varies depending upon which ligand was used, and is as indicated in Fig. 12 ▸.

## 4. Discussion   

Both mechanochemical methods and liquid diffusion syntheses, starting from cadmium(II) halide salts and the relevant ligands, resulted in one-dimensional coordination polymers as simple metal-based building units containing a central Cd—*X* structural spine equipped with *trans*-positioned bi-functional ligands. We succeeded in generating this structural core in eight out of nine synthetic attempts. Only the reaction of cadmium(II) iodide and quinazolinone yielded a monomeric tetrahedral complex, **9**. The complex also exhibits a uniquely observed coordination mode of the ligand within the studied series of complexes: it coordinates *via* the nitro­gen atom *ortho* to the carbonyl group. None of our alternative synthetic efforts (neither a solvothermal and mechanochemical synthetic procedure, nor supramolecular protection of the undesirable binding site) resulted in a structure with metal coordination *via* the nitro­gen atom positioned *meta* or *para* to the carbonyl group, as was observed in all other complexes **1**–**8**.

Even though our control of the details of the coordination chemistry in this series was very satisfying [a success rate of 89% (8/9) in preparing one-dimensional metal-containing polymeric chains], at the supramolecular level the desired connectivity was accomplished with a 100% success rate. In all examined compounds the basic building units were interconnected *via* the targeted primary type of non-covalent interaction, the single-point N—H⋯O hydrogen bond. Neither the dimensionality of the building block nor the coordination mode of the ligands prevented the appearance of this hydrogen bond. Moreover, even the halide anions, common counter-ions in metallo-supramolecular systems that are potentially disruptive to synthons which have already been well established in metal-free systems, did not exert any detrimental influence on the supramolecular synthesis. Generally, they carry a substantial negative charge that enables them to compete with hydrogen-bond acceptors that are already present as a part of the supramolecular functionality. In addition, the fact that they are also engaged as metal-bridging ligands reduces their overall negative charge to some extent, which should suppress their ability to compete for hydrogen-bond donors in the system. As a result, the required and intended N—H⋯O hydrogen bonds remained in place in all nine cases, suggesting that the C=O group is the most effective hydrogen-bond acceptor in these compounds.

To give an interpretation of the supramolecular observation, we calculated MEP surfaces (Hunter, 2004 ▸) in order to rank potential hydrogen-bond donors and acceptors according to the MEP values (Etter, 1990 ▸, 1991 ▸). In all nine cases, the carbonyl oxygen atom and the hydrogen atom attached to the endocyclic nitro­gen atom display the most negative and the most positive electrostatic potential value, respectively. Therefore, we can consider the two sites as the best hydrogen-bond acceptor and donor, respectively (Aakeröy & Kanisha, 2014 ▸). The fact that the preferred interaction systematically occurs between these two sites suggests that the ‘best donor⋯best acceptor’ guideline (Etter, 1990 ▸) can also be applied to carefully tailored metal–organic systems.

According to structural information extracted from the CSD, the supramolecular ‘driver’ used in this study (formed by having the carbonyl group directly adjacent to a heterocyclic nitro­gen atom) is predisposed to form dimers in metal-free systems. However, in our metal–organic systems the cyclic 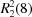 motif occurs in only two out of nine cases, while the catemeric *C*(4) motif dominates and appears in six out of nine cases. This is, in effect, an example of synthon crossover (Aakeröy *et al.*, 2011 ▸), which requires further examination.

**Figure 13:**
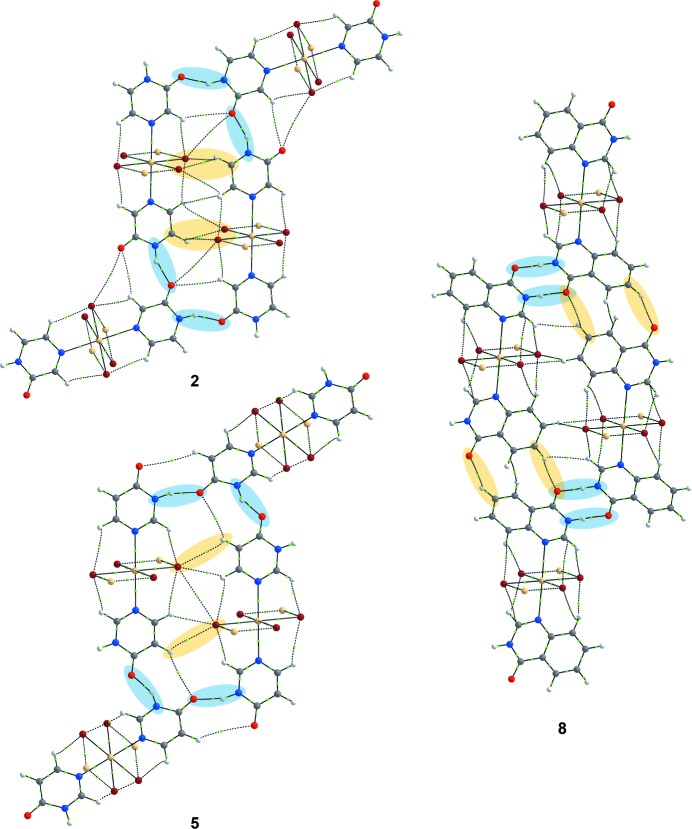
QTAIM analysis for a representative of each sub-class of complexes [CdBr_2_(2-pyz)_2_]*_n_* (**2**), [CdBr_2_(4-pym)_2_]*_n_* (**5**) and [CdBr_2_(4-quz)_2_]*_n_* (**8**), showing the bond-critical points as green dots. Interactions between the best donors and best acceptors are marked with blue ovals, while interactions involving the second-best donors are marked with yellow ovals. QTAIM analysis results for all the complexes can be found in the supporting information (Figs. S16–S19).

To better understand the reasons for this synthon crossover we first performed a QTAIM analysis. While both supra­molecular motifs, 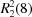 and *C*(4), contain the same number of N—H⋯O interactions per molecule (polymeric unit), the analysis showed that all crystal structures involving the catemeric *C*(4) motif (**1**–**6**) also display an additional ‘second-best donor⋯second-best acceptor’ interaction (C—H⋯*X*, Fig. 13 ▸), which is not present in structures dominated by the 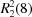 synthon. Instead, the second-best donor in those assemblies participates only in an interaction with the remaining lone pair of the best hydrogen-bond acceptor, namely the carbonyl oxygen atom.

**Figure 14:**
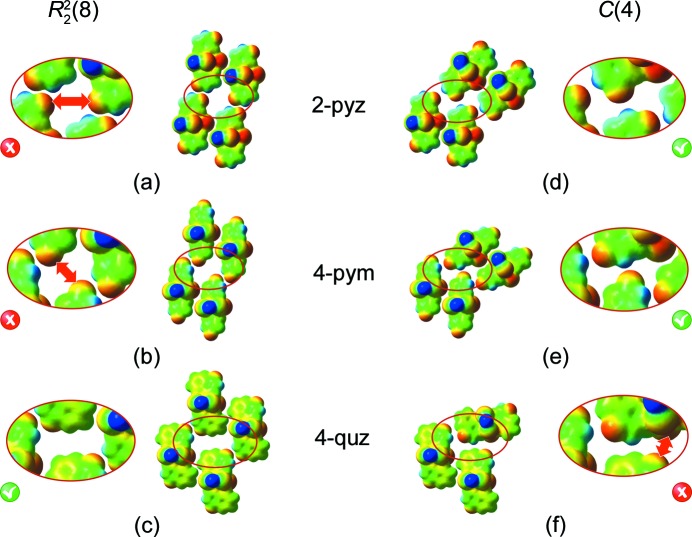
The observed (green ticks) and hypothetical (red crosses) models (assembled starting from one-dimensional coordination polymers represented by one metal centre for clarity) of the pyrazinone- (in compounds **1**–**3**), pyrimidinone- (in compounds **4**–**6**) and quinazolinone-based units (in compounds **7** and **8**) of the single-coordinated cadmium centre for compounds **2**, **5** and **8**, respectively.

Furthermore, for the three sub-classes of complexes in this series (Fig. 14 ▸) we compared the two outcomes, the dimeric and catemeric ones, to try to discern if any other close contacts would favour one particular assembly or synthon formation. If the 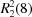 motif were to occur for the pyrazinone- (**1–3**) and pyrimidinone-containing (**4–6**) complexes, it would result in a much closer positioning of the two carbonyl O atoms (shown as red regions in the encircled part of Figs. 14 ▸ *a* and 14 ▸ *b*) compared with those in the observed structures (Figs. 14 ▸ *d* and 14 ▸ *e*), and this in turn would lead to increased electrostatic repulsion. In contrast, when the quinazolinone ligands are involved, the added spatial requirements of the second fused aromatic ring ensures that the two carbonyl oxygen atoms are separated when forming the 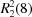 motif (Fig. 14 ▸ *c*). In addition, there are also stabilizing short contacts with hydrogen atoms from the ‘counter-surface area’ (green to pale-blue region, Fig. 14 ▸ *c* and supplementary Fig. S19). In the case of forming the catemeric motif *C*(4), again unfavourable contacts are to be expected (Fig. 14 ▸ *f*).

This detailed inspection of secondary hydrogen-bond interactions and of specific steric requirements clearly indicates that synthon crossover is controlled by a fine balance between close packing and the secondary hydrogen bonds that can form once the primary hydrogen-bond sites have been satisfied.

## 5. Conclusions   

Within the chemical and materials sciences, considerable efforts and resources are focused on elucidating structure–function correlations in order to understand the direct relationship between the relative arrangement of atoms and molecules and the properties that they display as a collective. However, much less work has been dedicated to developing reliable supramolecular synthetic pathways for actually organizing molecular building blocks into extended architectures with specific dimensionalities and topologies.

In this comprehensive study we combined synthesis, structural chemistry and cheminformatics with complementary theoretical tools in order to establish if crystal engineering principles that originate in the organic solid state can be adapted to the unique requirements and challenges posed by supramolecular synthesis in the coordination chemistry arena. The transition from organic to inorganic crystal engineering was accomplished through the design and structural implementation of ‘bi-functional’ ligands that are simultaneously capable of (i) interacting with metal ions in such a way that their coordination number and geometry remain invariant and predictable, and (ii) engaging in self-complementary hydrogen bonds that are highly directional and relatively insensitive to potentially interfering or competing sites. The main supra­molecular products that were targeted in this systematic study appeared in each and every one of the nine reported structures, which represents a very satisfactory outcome.

In order to test the durability of the synthetic strategy, we subsequently explored drastically different reaction conditions (solution-phase synthesis *versus* solvent-assisted grinding), but on no occasion did we observe a structurally different product, which emphasizes the robustness of the synthetic protocol.

The choice of self-complementary and catemeric synthons (responsible for the supramolecular assembly) in this study was guided by a simplified electrostatic view of hydrogen-bond interactions, and the results from the calculated MEP values showed that synthon transferability from organic to metal–organic systems is possible, since the relative importance and ranking of the different hydrogen-bond donors/acceptors remained the same even after the introduction of metal cations and charge-balancing anions. These results further support the hypothesis that a synthetic protocol built upon (i) the ‘best donor⋯best acceptor’ guidelines and (ii) a ranking of potential hydrogen-bonding acceptors/donors on the basis of MEP values (which has found considerable value in organic crystal engineering) can be successfully implemented for directing the effective supramolecular assembly of metal–organic systems. We expect that many more synthons and reproducible molecular recognition events that have initially been identified and explored in the organic solid state can be employed as synthetic vectors for the hierarchical assembly of metal-containing materials of considerable complexity and across a variety of length scales.

## Supplementary Material

Crystal structure: contains datablock(s) 1, 2, 3, 5, 6, 8, 9. DOI: 10.1107/S2052252517015494/lq5009sup1.cif

Structure factors: contains datablock(s) 1. DOI: 10.1107/S2052252517015494/lq50091sup2.hkl

Structure factors: contains datablock(s) 2. DOI: 10.1107/S2052252517015494/lq50092sup3.hkl

Structure factors: contains datablock(s) 3. DOI: 10.1107/S2052252517015494/lq50093sup4.hkl

Structure factors: contains datablock(s) 5. DOI: 10.1107/S2052252517015494/lq50095sup5.hkl

Structure factors: contains datablock(s) 6. DOI: 10.1107/S2052252517015494/lq50096sup6.hkl

Structure factors: contains datablock(s) 8. DOI: 10.1107/S2052252517015494/lq50098sup7.hkl

Structure factors: contains datablock(s) 9. DOI: 10.1107/S2052252517015494/lq50099sup8.hkl

Additional information, tables and figures. DOI: 10.1107/S2052252517015494/lq5009sup9.pdf

CCDC references: 1559137, 1559138, 1559139, 1559140, 1559141, 1559142, 1559143

## Figures and Tables

**Table 1:** Hydrogen-bond geometries for compounds **1**–**3**, **5**, **6**, **8** and **9**

Compound	*D*—H⋯*A*	*D*⋯*A* (Å)	*D*—H⋯*A* (°)
**1**	N2—H21⋯O1^i^	2.712 (8)	160 (8)
	C1—H1⋯Cl1^ii^	3.446 (8)	123.1
	C3—H3⋯Cl1^iii^	3.512 (7)	141.5
	C4—H4⋯Cl1^iv^	3.500 (8)	130.2
			
**2**	N2—H21⋯O1^v^	2.72 (1)	155 (10)
	C1—H1⋯Br1^vi^	3.542 (9)	125.9
	C3—H3⋯Br1^vii^	3.659 (9)	137.8
	C4—H4⋯Br1^viii^	3.596 (9)	132.6
			
**3**	N2—H21⋯O1^v^	2.714 (6)	161 (5)
	C1—H1⋯I1^vii^	3.703 (5)	128.2
	C4—H4⋯I1^viii^	3.789 (5)	136.0
	C3—H3⋯I1^vii^	3.896 (5)	133.8
			
**5**	N2—H21⋯O1^ix^	2.750 (8)	154 (7)
	C1—H1⋯Br1	3.521 (7)	133.8
	C3—H3⋯Br1^x^	3.824 (7)	122.9
			
**6**	N2—H21⋯O1^x^	2.74 (2)	163 (14)
	C1—H1⋯I1^xi^	3.66 (1)	125.6
	C3—H3⋯I1^xii^	4.10 (1)	126.1
			
**8**	N2—H21⋯O1^xii^	2.76 (1)	175.5
	C1—H1⋯Br1^xiii^	3.571 (7)	130.8
	C5—H5⋯O1^xi^	3.57 (1)	114.0
			
**9**	N2—H21⋯O1^xv^	2.930 (6)	173 (6)

Symmetry codes: (i) 
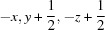
; (ii) 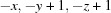; (iii) 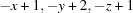; (iv) 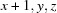; (v) 
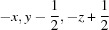
; (vi) 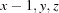; (vii) 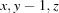; (viii) 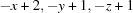; (ix) 
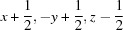
; (x) 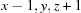; (xi) 
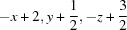
; (xii) 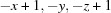; (xiii) 
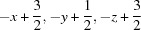
; (xiv) 
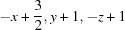
; (xv) 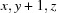.

**Table 2:** Calculated MEP values (in kJ mol^−1^) on the two best hydrogen-bond donor and acceptor sites for compounds **1**–**9**, as indicated in Fig. 12 ▸ (blue and red atoms)

Compound	O	*X*	H(N)	H(C)
**1**	−215	−181	298	225
**2**	−212	−150	301	226
**3**	−200	−120	296	220
**4**	−188	−116	305	143
**5**	−188	−90	310	140
**6**	−187	−61	311	141
**7** †	−205	−120	256	111
**8**	−200	−97	256	112
**9** ‡	−193	−78	252	109

† NALFEN.

‡ Hypothetical model of **9** if it were isostructural with **7** and **8**.
