# Supplementary material for: Building inorganic supramolecular architectures using principles adopted from the organic solid state
Source: IUCrJ. 2018 Jan 1;5(Pt 1):13–21. doi: 10.1107/S2052252517015494 (PMC5755573; doi:10.1107/S2052252517015494)
Supplement: Supplementary file 9 [file m-05-00013-sup9.pdf]

IUCrJ

Volume 5 (2018)

Supporting information for article:

Building inorganic supramolecular architectures using principles adopted from the organic solid state

Marijana Đaković, Željka Soldin, Boris-Marko Kukovec, Ivan Kodrin, Christer B. Aakeröy, Nea Baus and Tamara Rinkovec

S1. Experimental

S1.1. Methods

CHN analyses were performed with a Perkin-Elmer 2400 Series II CHNS analyzer in the Analytical Services Laboratories of the Ruđer Bošković Institute, Zagreb, Croatia.

IR analyses were performed on a PerkinElmer Spectrum Two spectrometer equipped with Diamond UATR accessory. FT-IR spectra were measured in ATR mode in the range 4000–450 cm^{-1} with resolution 2 cm^{-1}.

Thermogravimetric measurements were performed using a simultaneous TGA-DTA analyzer (Mettler-Toledo TGA/SDTA 851e). The samples were placed in aluminum pans (40 µL), heated in flowing nitrogen (120 mL min^{-1}) from room temperature up to 600 °C at a rate of 10 °C min^{-1}. Data collection and analysis were performed using the program package STARe Software 9.01 (MettlerToledo GmbH, 2006.).

X-Ray powder diffraction experiments were performed on a Philips PW 1850 diffractometer, CuKα radiation, voltage 40 kV, and current 40 mA. The patterns were collected in the angle region between 4° and 50° (2θ) with a step size of 0.02°.

S1.2. Synthesis

[CdCl$_2$(2-pyz)$_2$]$_n$, 1. Used: $CdCl_2$ (18.3 mg, 0.1 mmol) and 2(1*H*)-pyrazinone (2-pyz) (19.2 mg, 0.2 mmol). Yield: 73 % (27.4 mg). *Microanalysis*. Calc. for $C_8H_8N_4O_2CdCl_2$ (M_r = 375.49): C, 25.59; H, 2.15; N, 14.92. Found: C, 25.16; H, 1.97; N, 14.72 %. ATR-FTIR (cm^{-1}): 1722 w, 1680 vs (ν(C=O)), 1608 vs (C=N and C=C in plane ring vibration). Thermal analysis: decomposition occurs in three steps, the first one between 164 and 294 °C (endothermic DTA peak at 276 °C); the second one between 295 and 449 °C (endothermic DTA peak at 355 °C); further decomposition is observed between 450 and 600 °C (endothermic DTA peak at 473 °C); after heating up to 600 °C 51 % of initial mass remains.

The powder diffraction pattern (bulk sample) was consistent with the pattern calculated from single crystal data (Fig. S4).

Mechanochemical synthesis. Used: $CdCl_2$ (73.3 mg; 0.4 mmol) and 2(1*H*)-pyrazinone (2pyz) (76.9 mg; 0.8 mmol). *Microanalysis*. Calc. for $C_8H_8N_4O_2CdCl_2$ (M_r = 375.49): C, 25.59; H, 2.15; N, 14.92. Found: C, 25.21; H, 2.01; N, 14.11%. The powder diffraction pattern matched with that of **1** prepared by the solution method (Fig. S4).

$[CdBr_2(2\text{-pyz})_2]_n$, 2. Used: $CdBr_2{\cdot}4H_2O$ (34.4 mg, 0.1 mmol) and 2(1*H*)-pyrazinone (2-pyz) (19.2 mg, 0.2 mmol). Yield: 80 % (37.2 mg). *Microanalysis.* Calc. for $C_8H_8N_4O_2CdBr_2$ (*Mr* = 464.40): C, 20.69; H, 1.74; N, 12.07. Found: C, 20.54; H, 1.34; N, 11.97 %. ATR-FTIR (cm^{-1}): 1721 w, 1674 vs (ν(C=O)), 1603 s (C=N and C=C in plane ring vibration). Thermal analysis: decomposition occurs in three steps, the first one between 219 and 282 °C (endothermic DTA peak at 276 °C); the second one between 283 and 314 °C (endothermic DTA peak at 303 °C); further decomposition is observed between 424 and 600 °C (endothermic DTA peak at 563 °C); after heating up to 600 °C 14 % of initial mass remains.

The powder diffraction pattern (bulk sample) was consistent with the pattern calculated from single crystal data (Fig. S5).

Mechanochemical synthesis. Used: $CdBr_2{\cdot}4H_2O$ (137.7 mg; 0.4 mmol) and 2(1*H*)-pyrazinone (2-pyz) (76.9 mg; 0.8 mmol). *Microanalysis.* Calc. for $C_8H_8N_4O_2CdBr_2$ (*Mr* = 464.40): C, 20.69; H, 1.74; N, 12.07. Found: C, 20.09; H, 1.26; N, 11.89%. The powder diffraction pattern matched with that of **2** prepared by the solution method (Fig. S5).

$[CdI_2(2\text{-pyz})_2]_n$, 3. Used: CdI_2 (36.6 mg, 0.1 mmol) and 2(1*H*)-pyrazinone (2-pyz) (19.2 mg, 0.2 mmol). Yield: 78 % (43.6 mg). *Microanalysis.* Calc. for $C_8H_8N_4O_2CdI_2$ (M_r = 558.40): C, 17.21; H, 1.44; N, 10.03. Found: C, 16.88; H, 1.26; N, 10.13 %. ATR-FTIR (cm^{-1}): 1715 w, 1675 s (ν(C=O)), 1599 s (C=N and C=C in plane ring vibration). Thermal analysis: decomposition occurs in two steps, the first one between 160 and 223 °C (endothermic DTA peak at 208 °C); further decomposition is observed between 224 and 600 °C; after heating up to 600 °C 48% of initial mass remains.

The powder diffraction pattern (bulk sample) was consistent with the pattern calculated from single crystal data (Fig. S6).

Mechanochemical synthesis. Used: CdI_2 (146.5 mg; 0.4 mmol) and 2(1*H*)-pyrazinone (2-pyz) (76.9 mg; 0.8 mmol). Calc. for $C_8H_8N_4O_2CdI_2$ (M_r = 558.40): C, 17.21; H, 1.44; N, 10.03. Found: C, 17.37; H, 1.12; N, 9.83 %. The powder diffraction pattern matched with that of **3** prepared by the solution method (Fig. S6).

$[CdCl_2(4\text{-pym})_2]_n$, 4. Used: $CdCl_2$ (18.3 mg, 0.1 mmol) and 4(3*H*)-pyrimidinone (4-pym) (19.2 mg, 0.2 mmol). Yield: 84 % (31.5 mg). *Microanalysis*. Calc. for $C_8H_8N_4O_2CdCl_2$ (M_r = 375.49): C, 25.59; H, 2.15; N, 14.92. Found: C, 25,40; H, 1,27; N, 14,81 %. ATR-FTIR (cm^{-1}): 1728 s, 1702 s (ν(C=O)), 1600 vs (C=N and C=C in plane ring vibration). Thermal analysis: decomposition occurs in three steps, the first one between 175 and 288 °C (endothermic DTA peak at 279 °C); the second one between 289 and 395 °C (endothermic DTA peak at 336 °C); further decomposition is observed

between 426 and 600 °C (endothermic DTA peak at 483 °C); after heating up to 600 °C 51 % of initial mass remains.

The powder diffraction pattern (bulk sample) was consistent with the pattern calculated from single crystal data (Fig. S8).

Mechanochemical synthesis. Used: $CdCl_2$ (73.3 mg; 0.4 mmol) and 4(3*H*)-pyrimidinone (4-pym) (76.9 mg; 0.8 mmol). *Microanalysis*. Calc. for $C_8H_8N_4O_2CdCl_2$ (M_r = 375.49): C, 25.59; H, 2.15; N, 14.92. Found: C, 25.79; H, 2.07; N, 14.63%. The powder diffraction pattern matched with that of **4** prepared by the solution method (Fig. S8).

$[CdBr_2(4\text{-pym})_2]_n$, 5. Used: $CdBr_2\cdot 4H_2O$ (34.4 mg, 0.1 mmol) and 4(3*H*)-pyrimidinone (4-pym) (19.2 mg, 0.2 mmol). Yield: 77 % (35.8 mg). *Microanalysis.* Calc. for $C_8H_8N_4O_2CdBr_2$ (*Mr* = 464.40): C, 20.69; H, 1.74; N, 12.07. Found: C, 19,69; H, 1,07; N, 11,38%. ATR-FTIR (cm^{-1}): 1721 m, 1699 m (ν(C=O)), 1597 s (C=N and C=C in plane ring vibration). Thermal analysis: decomposition occurs in three steps, the first one between 245 and 312 °C (endothermic DTA peak at 303 °C); the second one between 313 and 359 °C (endothermic DTA peak at 321 °C); further decomposition is observed between 360 and 600 °C; after heating up to 600 °C 56 % of initial mass remains.

The powder diffraction pattern (bulk sample) was consistent with the pattern calculated from single crystal data (Fig. S9).

Mechanochemical synthesis. Used: $CdBr_2\cdot 4H_2O$ (137.7 mg; 0.4 mmol) and 4(3*H*)-pyrimidinone (4-pym) (76.9 mg; 0.8 mmol). *Microanalysis.* Calc. for $C_8H_8N_4O_2CdBr_2$ (*Mr* = 464.40): C, 20.69; H, 1.74; N, 12.07. Found: C, 20.23; H, 1.65; N, 11.96 %. The powder diffraction pattern matched with that of **5** prepared by the solution method (Fig. S9).

$[CdI_2(4\text{-pym})_2]_n$, 6. Used: CdI_2 (36.6 mg, 0.1 mmol) and 4(3*H*)-pyrimidinone (4-pym) (19.2 mg, 0.2 mmol). Yield: 81 % (45.3 mg). *Microanalysis.* Calc. for $C_8H_8N_4O_2CdI_2$ (M_r = 558.40): C, 17.21; H, 1.44; N, 10.03. Found: C, 17.04; H, 1.26; N, 9.73 %. ATR-FTIR (cm^{-1}): 1722 m, 1693 s (ν(C=O)), 1599 s (C=N and C=C in plane ring vibration). Thermal analysis: endothermic DTA peak due to the melting of the compound is observed at 187 °C; decomposition occurs in two steps, the first one between 220 and 320 °C (endothermic DTA peak at 280 °C); further decomposition is observed between 321 and 600 °C; after heating up to 600 °C 38 % of initial mass remains.

The powder diffraction pattern (bulk sample) was consistent with the pattern calculated from single crystal data (Figs. S10).

Mechanochemical synthesis. Used: CdI_2 (146.5 mg; 0.4 mmol) and 4(3*H*)-pyrimidinone (4-pym) (76.9 mg; 0.8 mmol). Calc. for $C_8H_8N_4O_2CdI_2$ (M_r = 558.40): C, 17.21; H, 1.44; N, 10.03. Found: C, 17.13; H, 1.31; N, 9.76 %.The powder diffraction pattern matched with that of **6** prepared by the solution method (Fig. S10).

[$CdCl_2$(4-quz)$_2$]$_n$, 7. ***Mechanochemical synthesis***. Used: $CdCl_2$ (73.3 mg; 0.4 mmol) and 4(3*H*)-quinazolinone (4-quz) (116.9 mg; 0.8 mmol). *Microanalysis.* Calc. for $C_8H_8N_4O_2CdCl_2$ (*Mr* = 375.49): C, 25.59; H, 2.15; N, 14.92. Found: C, 25.22; H, 1.81; N, 14.51 %. The powder diffraction pattern matched with that of **7** prepared by the solution method and with the pattern calculated from single crystal data (Fig. S12).

[$CdBr_2$(4-quz)$_2$]$_n$, 8. Used: $CdBr_2\cdot4H_2O$ (34.4 mg, 0.1 mmol) and 4(3*H*)-quinazolinone (4-quz) (29.2 mg, 0.2 mmol). Yield: 84% (47.4 mg). *Microanalysis.* Calc. for $C_{16}H_{12}N_4O_2CdBr_2$ (*Mr* = 564.52): C, 34.04; H, 2.14; N, 9.93. Found: C, 33.89; H, 1.38; N, 9.86 %. ATR-FTIR (cm^{-1}): 1690 vs (ν(C=O)), 1618 m (C=N and C=C in plane ring vibration). Thermal analysis: endothermic DTA peak due to the melting of the compound is observed at 241 °C; decomposition occurs in two steps, the first one between 243 and 367 °C (endothermic DTA peak at 346 °C); further decomposition is observed between 368 and 600 °C (endothermic DTA peak at 450 °C); after heating up to 600 °C 58 % of initial mass remains.

The powder diffraction pattern (bulk sample) was consistent with the pattern calculated from single crystal data (Fig. S13).

Mechanochemical synthesis. Used: $CdBr_2\cdot4H_2O$ (137.7 mg; 0.4 mmol) and 4(3*H*)- quinazolinone (4-quz) (116.9 mg; 0.8 mmol). *Microanalysis.* Calc. for $C_8H_8N_4O_2CdBr_2$ (*Mr* = 464.40): C, 20.69; H, 1.74; N, 12.07. Found: C, 20.49; H, 1.56; N, 11.97 %. The powder diffraction pattern matched with that of **7** prepared by the solution method (Fig. S13).

[CdI_2(4-quz)$_2$], 9. Used: CdI_2 (36.6 mg, 0.1 mmol) and 4(3*H*)- quinazolinone (4-quz) (29.2 mg, 0.2 mmol). Yield: 77 % (50.7 mg). *Microanalysis.* Calc. for $C_{16}H_{12}N_4O_2CdI_2$ (*Mr* = 658.51): C, 29.18; H, 1.84; N, 8.51. Found: C, 29.06; H, 1.70; N, 8.50 %. ATR-FTIR (cm^{-1}): 1690 vs (ν(C=O)), 1571 m (C=N and C=C in plane ring vibration). Thermal analysis: endothermic DTA peak due to the melting of the compound is observed at 227 °C; decomposition occurs in two steps, the first one between 230 and 500 °C (exothermic DTA peak at 336 °C); further decomposition is observed between 501 and 600 °C (endothermic DTA peak at 543 °C); after heating up to 600 °C 52 % of initial mass remains.

The powder diffraction pattern (bulk sample) was consistent with the pattern calculated from single crystal data (Fig. S14).

Mechanochemical synthesis. Used: CdI_2 (146.5 mg; 0.4 mmol) and 4(3*H*)- quinazolinone (4-quz) (116.9 mg; 0.8 mmol). Calc. for $C_{16}H_{12}N_4O_2CdI_2$ (Mr = 658.51): C, 29.18; H, 1.84; N, 8.51. Found: C, 28.98; H, 1.36; N, 8.42 %. The powder diffraction pattern matched with that of **8** prepared by the solution method (Fig. S14).

S2. Single crystal X-ray crystallography

Table S1 Crystal data and details of the structure determination for **1−3**, **5–6, 8–9**.

Compound	**1**	**2**	**3**	**5**
Formula	$C_8H_8CdCl_2N_4O_2$	$C_8H_8CdBr_2N_4O_2$	$C_8H_8CdI_2N_4O_2$	$C_8H_8CdBr_2N_4O_2$
M_r	375.48	464.40	558.38	464.40
Colour and habit	colourless, prism	colourless, prism	colourless, prism	colourless, prism
Crystal system, space group	monoclinic, $P2_1/c$ (No. 14)	monoclinic, $P2_1/c$ (No. 14)	monoclinic, $P2_1/c$ (No. 14)	monoclinic, $P2_1/n$ (No. 14)
Crystal dimensions (mm^3)	0.82 × 0.14 × 0.10	0.34 × 0.24 × 0.16	0.60 × 0.18 × 0.14	0.80 × 0.10 × 0.06
a (Å)	3.7833(3)	3.9043(2)	4.1497(2)	3.8840(3)
b (Å)	7.6139(7)	7.7180(5)	7.8783(4)	23.164(2)
c (Å)	20.1473(18)	20.6142(14)	21.0091(14)	7.0359(8)
α (˚)	90	90	90	90
β (˚)	92.131(7)	92.442(5)	92.551(4)	93.712(9)
γ (˚)	90	90	90	90
V (Å^3)	579.96(9)	620.61(7)	686.16(7)	631.67(10)
Z	2	2	2	2
D_{calc} (g cm^{-3})	2.150	2.485	2.703	2.442
μ (mm^{-1})	2.337	8.192	6.086	8.049
F(000)	364	436	508	436
θ range for data collection (˚)	4.86–24.99	4.76–25.00	4.67–24.99	4.56–24.99

h,*k*,*l* range	-3:4, -9:9, -23:23	-4:4, -8:9, -24:24	-4:4, -5:9, -24:24	-4:4, -14:27, -8:7
Scan type	ω	ω	ω	ω
No. measured reflections	4107	3352	2305	1940
No. independent reflections (R_{int})	1020 (0.0357)	1092 (0.0189)	1198 (0.0167)	1108 (0.0284)
No. observed reflections, $I \geq 2\sigma(I)$	961	1037	1121	944
No. refined parameters	82	83	82	82
R, wR [$I \geq 2\sigma(I)$]	0.0470, 0.1074	0.0384, 0.0989	0.0254, 0.0574	0.0445, 0.0962
R, wR [all data]	0.0492, 0.1080	0.0397, 0.0993	0.0275, 0.0584	0.0550, 0.1012
Goodness of fit on F^2, S	1.531	1.376	1.187	1.147
Max., min. electron density (e Å^{-3})	1.55, -0.74	1.76, -0.87	0.73, -0.62	1.67, -0.79
CCDC number	1559137	1559138	1559139	1559140

Compound	**6**	**8**	**9**
Formula	$C_8H_8CdI_2N_4O_2$	$C_{16}H_{12}CdBr_2N_4O_2$	$C_{16}H_{12}CdI_2N_4O_2$
M_r	558.38	564.52	658.50
Colour and habit	colourless, prism	colourless, prism	colourless, prism
Crystal system, space group	monoclinic, $P2_1/c$ (No. 14)	monoclinic, *I*2/*a* (No. 15)	monoclinic, *I*2/*a* (No. 15)
Crystal dimensions (mm^3)	0.42 × 0.14 × 0.06	0.76 × 0.07 × 0.06	0.30 × 0.19 × 0.14
a (Å)	4.0953(3)	18.2590(12)	19.7099(5)
b (Å)	7.8089(6)	3.8670(2)	6.8535(2)
c (Å)	21.7078(13)	24.2737(18)	13.6310(4)
α (˚)	90	90	90
β (˚)	92.307(6)	95.491(6)	98.052(3)
γ (˚)	90	90	90
V (Å^3)	693.65(8)	1706.04(19)	1823.15(9)
Z	2	4	4
D_{calc} (g cm^{-3})	2.673	2.198	2.399
μ (mm^{-1})	6.020	5.982	4.602
F(000)	508	1080	1224
θ range for data collection (˚)	4.58–26.97	4.23–26.99	4.18–25.00
h,*k*,*l* range	-5:4, -4:9, -13:27	-23:22, -4:4, -30:27	-23:23, -8:4, -16:16
Scan type	ω	ω	ω
No. measured reflections	2578	7204	5658
No. independent reflections (R_{int})	1480 (0.0260)	1841 (0.0488)	1596 (0.0294)
No. observed reflections, $I \geq 2\sigma(I)$	1368	1446	1439
No. refined parameters	83	115	117
R, w*R* [$I \geq 2\sigma(I)$]	0.0501, 0.1437	0.0551, 0.1629	0.0382, 0.0936

R, w*R* [all data]	0.0556, 0.1491	0.0690, 0.1769	0.0431, 0.0980
Goodness-of-fit on F^2, *S*	1.171	1.076	1.086
Max., min. electron density (e $Å^{-3}$)	3.59, -1.38	3.64, -0.76	3.46, -0.65
CCDC number	1559141	1559142	1559143

Table S2 Selected bond distances (Å) and angles (°) for **1−3**, **5–6, 8–9**.

**1**		**2**		**5**	
Bond distances					
Cd1–N1	2.411(6)	Cd1–N1	2.455(7)	Cd1–N1	2.404(6)
Cd1–Cl1	2.600(2)	Cd1–Br1	2.7098(9)	Cd1–Br1	2.7498(7)
Cd1–Cl1^{i}	2.617(2)	Cd1–Br1^{iv}	2.7585(9)	Cd1–Br1^{iv}	2.7507(8)
Bond angles					
N1–Cd1–Cl1	86.6(2)	N1–Cd1–Br1	93.1(2)	N1–Cd1–Br1	89.5(1)
N1–Cd1–Cl1^{ii}	93.5(2)	N1–Cd1–Br1^{ii}	86.9(2)	N1–Cd1–Br1^{vi}	90.5(1)
N1–Cd1–Cl1^{i}	90.8(2)	N1–Cd1–Br1^{iv}	89.2(2)	N1–Cd1–Br1^{iv}	88.9(1)
Cl1–Cd1–Cl1^{i}	92.96(6)	Br1–Cd1–Br1^{iv}	91.12(3)	Br1–Cd1–Br1^{iv}	89.84(2)
N1–Cd1–Cl1^{iii}	89.2(2)	N1–Cd1–Br1^{v}	90.8(2)	N1–Cd1–Br1^{vii}	91.2(1)
Cl1–Cd1–Cl1^{iii}	87.04(6)	Br1–Cd1–Br1^{v}	88.88(3)	Br1–Cd1–Br1^{vii}	90.16(2)

Symmetry codes (i): x+1, y, z; (ii): -x+1, -y+1, -z+1; (iii): -x, -y+1, -z+1; (iv): x-1, y, z; (v): -x+2, -y+1, -z+1; (vi): -x+1, -y, -z+1; (vii): -x+2, -y, -z+1

	**3**	**6**
Bond distances		
Cd1–N1	2.506(4)	2.429(9)
Cd1–I1	2.8729(3)	2.8916(7)
Cd1–I1^{iv}	2.9957(3)	3.0046(7)
Bond angles		
N1–Cd1–I1	93.46(9)	87.2(3)
N1–Cd1–I1^{ii}	86.54(9)	92.8(3)
N1–Cd1–I1^{iv}	89.39(9)	90.2(2)
I1–Cd1–I1^{iv}	89.975(9)	87.96(2)
N1–Cd1–I1^{v}	90.61(9)	89.8(2)
I1–Cd1–I1^{v}	90.03(1)	92.04(2)

Symmetry codes (ii): -x+1, -y+1, -z+1; (iv): x-1, y, z; (v): -x+2, -y+1, -z+1

8		9	
Bond distances			
Cd1–N1	2.482(6)	Cd1–N1	2.305(4)
Cd1–Br1	2.7474(8)	Cd1–I1	2.7207(5)
Cd1–Br1^{viii}	2.7040(8)		
Bond angles			
N1–Cd1–N1^{ix}	175.6(3)	N1^{xi}–Cd1–N1	102.6(2)
N1–Cd1–Br1^{viii}	94.6(2)	N1–Cd1–I1^{xi}	112.1(1)
N1–Cd1–Br1^{x}	88.5(2)	N1–Cd1–I1	105.9(1)
Br1^{viii}–Cd1–Br1^{x}	90.54(4)	I1^{xi}–Cd1–I1	117.32(3)
N1–Cd1–Br1^{ix}	85.4(2)		
N1–Cd1–Br1	91.4(2)		
Br1^{viii}–Cd1–Br1	90.36(2)		
Br1^{x}–Cd1–Br1	179.09(3)		
Br1^{ix}–Cd1–Br1	88.73(3)		

Symmetry code (viii): x, y+1, z; (ix): -x+3/2, y, -z+1; (x): -x+3/2, y+1, -z+1; (xi): -x+1/2, y, -z+1

Table S3 Hydrogen bond geometry for **1−3**, **5–6, 8–9**.

D–H···A	*d*(D–H)/Å	*d*(H···A)/Å	*d*(D···A)/Å	∠(D–H···A)/°	Symmetry code on A
**1**					
N2–H21···O1	0.88(2)	1.87(4)	2.712(8)	160(8)	-x, y+1/2, -z+1/2
C1–H1···Cl1	0.95	2.83	3.446(8)	123.1	-x, -y+1, -z+1
C3–H3···Cl1	0.95	2.72	3.512(7)	141.5	-x+1, -y+2, -z+1
C4–H4···Cl1	0.95	2.81	3.500(8)	130.2	x+1, y, z
**2**					
N2–H21···O1	0.86(2)	1.92(5)	2.72(1)	155(10)	-x, y-1/2, -z+1/2
C1–H1···Br1	0.93	2.92	3.542(9)	125.9	x-1, y, z
C3–H3···Br1	0.93	2.92	3.659(9)	137.8	x, y-1, z
C4–H4···Br1	0.93	2.90	3.596(9)	132.6	-x+2, -y+1, -z+1
**3**					
N2–H21···O1	0.86(2)	1.89(3)	2.714(6)	161(5)	-x, y-1/2, -z+1/2
C1–H1···I1	0.93	3.05	3.703(5)	128.2	x-1, y, z
C4–H4···I1	0.93	3.06	3.789(5)	136.0	-x+2, -y+1, -z+1
C3–H3···I1	0.93	3.19	3.896(5)	133.8	x, y-1, z
**5**					
N2–H21···O1	0.85(2)	1.96(4)	2.750(8)	154(7)	x+1/2, -y+1/2, z-1/2
C1–H1···Br1	0.93	2.81	3.521(7)	133.8	x, y, z
C3–H3···Br1	0.93	3.24	3.824(7)	122.9	x-1, y, z+1
**6**					
N2–H21···O1	0.88(2)	1.89(5)	2.74(2)	163(14)	-x+2, y+1/2, -z+3/2
C1–H1···I1	0.95	3.02	3.66(1)	125.6	-x+2, -y+1, -z+1
C3–H3···I1	0.95	3.47	4.10(1)	126.1	-x+1, -y, -z+1
**8**					
N2–H21···O1	0.88	1.88	2.76(1)	175.5	-x+3/2, -y+1/2, -z+3/2
C1–H1···Br1	0.95	2.88	3.571(7)	130.8	-x+3/2, y+1, -z+1

C5–H5···O1	0.95	3.08	3.57(1)	114.0	-x+2, y+1/2, -z+3/2
**9**					
N2–H21···O1	0.85(2)	2.08(2)	2.930(6)	173(6)	x, y+1, z

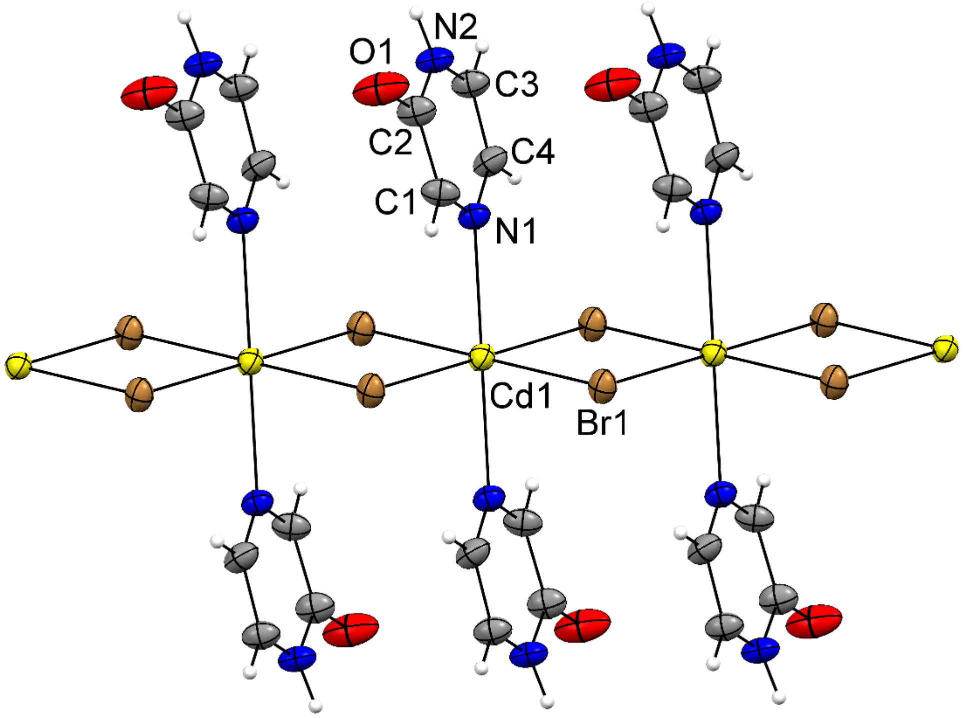

Figure S1 ORTEP-style plot of $[CdBr_2(2\text{-pyz})_2]_n$ (**2**), with a labelling scheme of the asymmetric unit. Thermal ellipsoids are drawn at 50% probability level at 296(2) K.

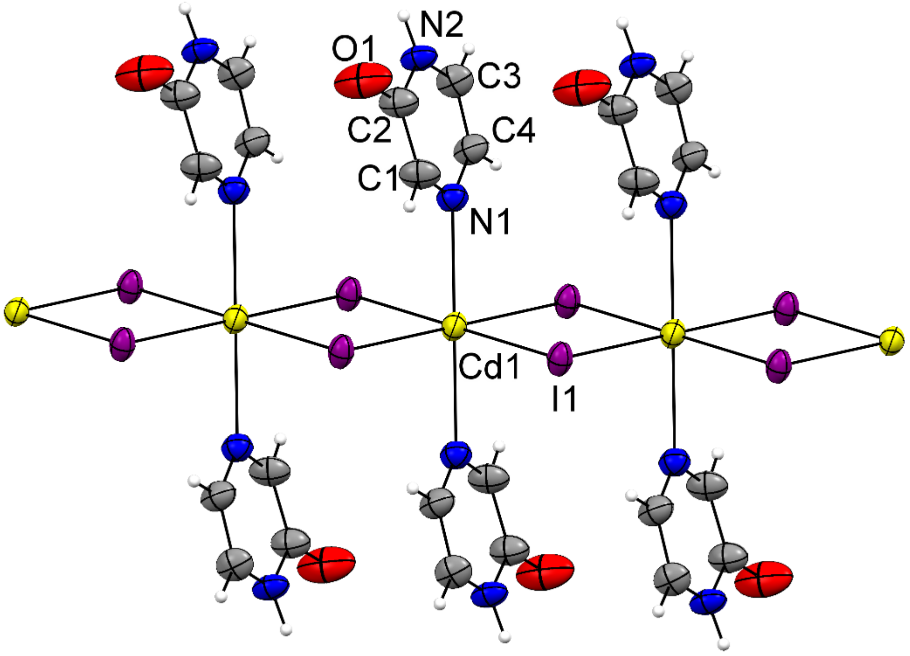

Figure S2 ORTEP-style plot of $[CdI_2(2\text{-pyz})_2]_n$ (**3**), with a labelling scheme of the asymmetric unit. Thermal ellipsoids are drawn at 50% probability level at 296(2) K.

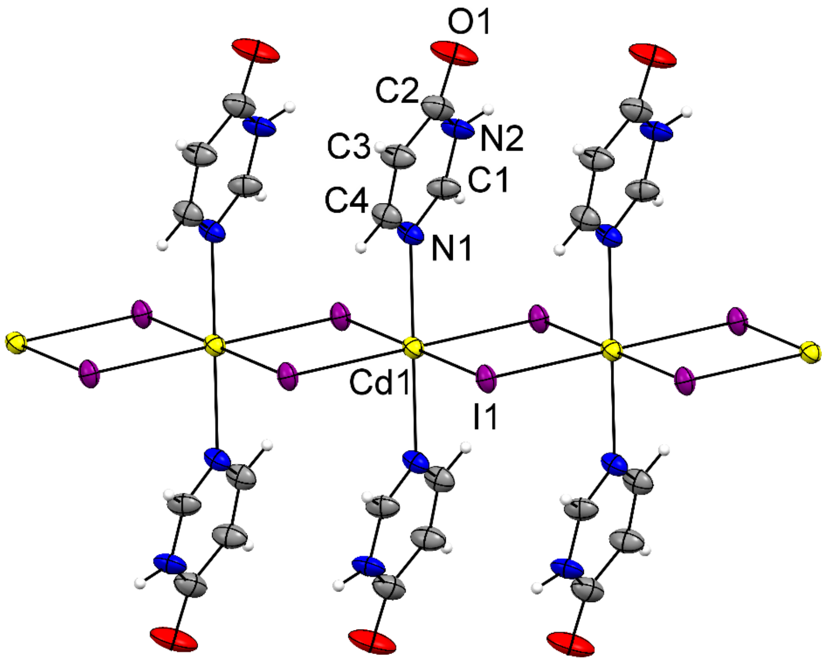

Figure S3 ORTEP-style plot of $[CdI_2(4\text{-pym})_2]_n$ (**6**), with a labelling scheme of the asymmetric unit. Thermal ellipsoids are drawn at 50% probability level at 200(2) K.

S3. Powder X-ray crystallography

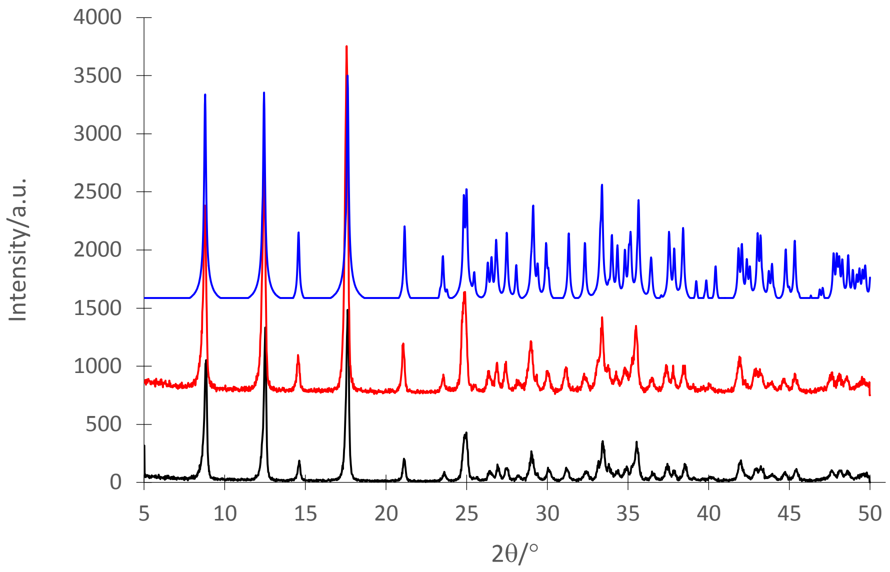

Figure S4 Experimental (solution – black; mechanochemical – red) and simulated (blue) PXRD traces of $[CdCl_2(2\text{-pyz})_2]_n$ (**1**)

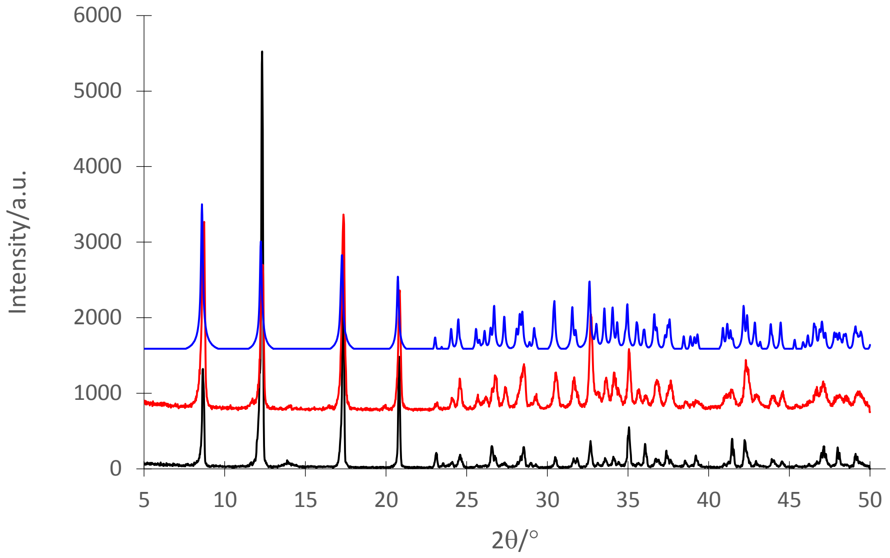

Figure S5 Experimental (solution – black; mechanochemical – red) and simulated (blue) PXRD traces of $[CdBr_2(2\text{-pyz})_2]_n$ (**2**)

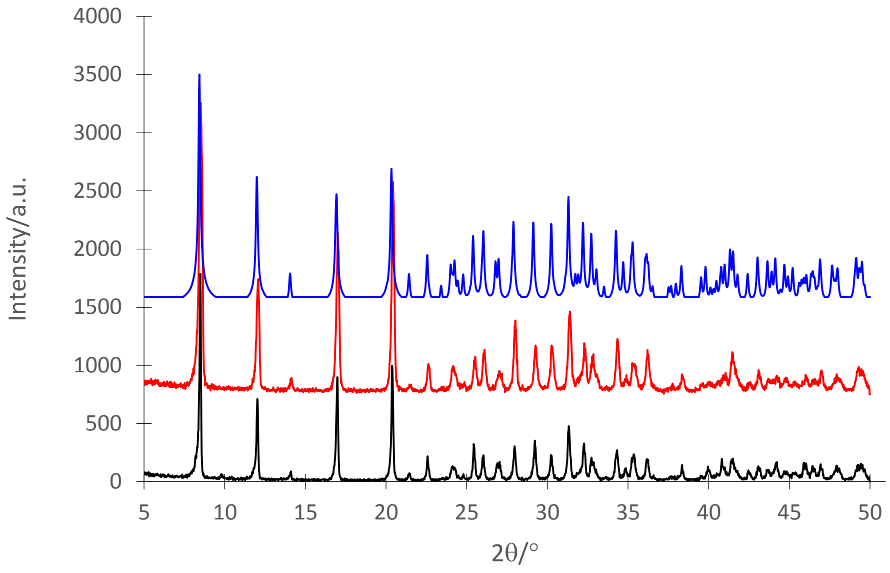

Figure S6 Experimental (solution – black; mechanochemical – red) and simulated (blue) PXRD traces of $[CdI_2(2\text{-pyz})_2]_n$ (**3**)

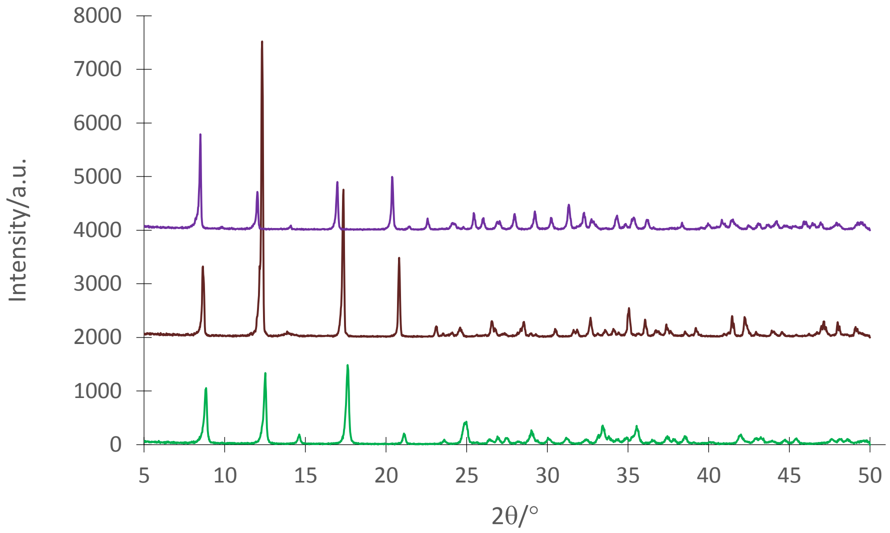

Figure S7 Overlay of experimental PXRD traces for $[CdCl_2(2\text{-pyz})_2]_n$ (**1**) (green), $[CdBr_2(2\text{-pyz})_2]_n$ (**2**) (brown) and $[CdI_2(2\text{-pyz})_2]_n$ (**3**) (violet).

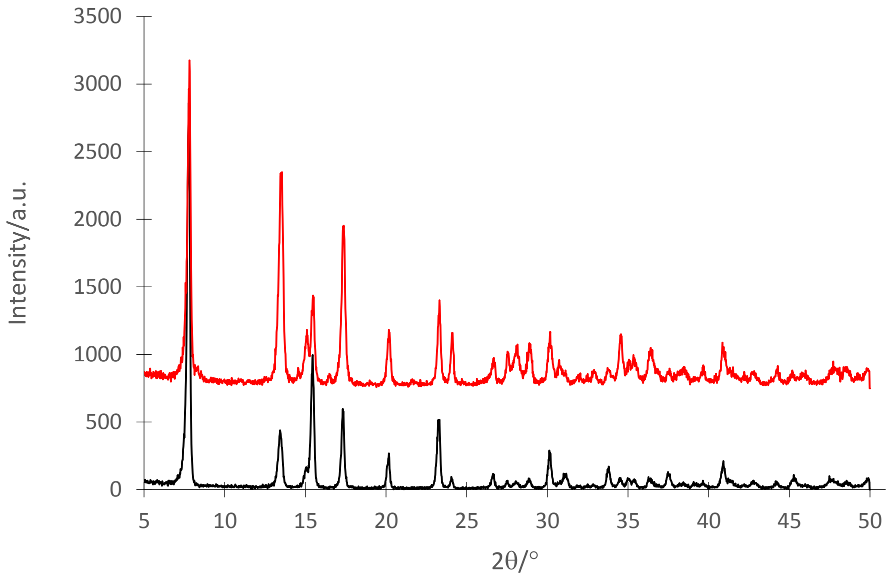

Figure S8 Experimental, solution (black) and mechanochemical (red) PXRD traces of $[CdCl_2(4\text{-}pym)_2]_n$ (**4**).

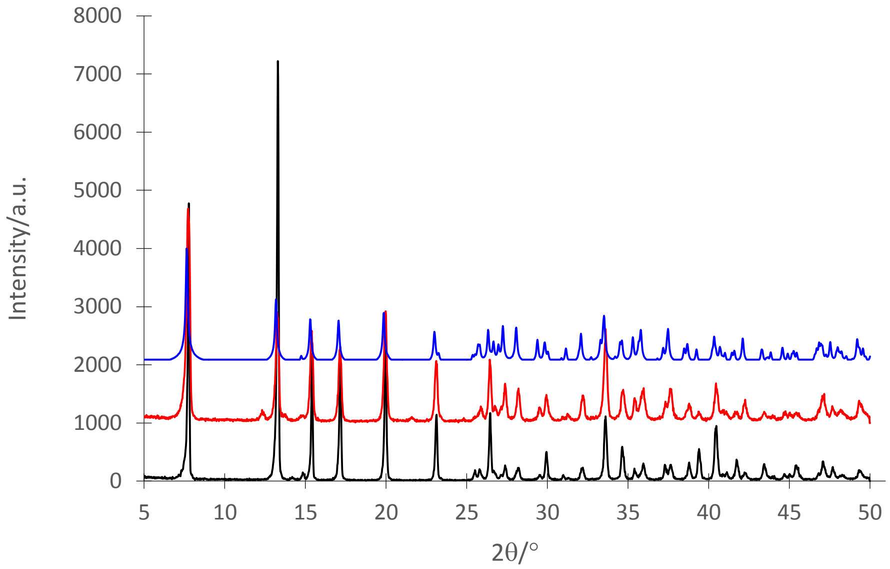

Figure S9 Experimental (solution – black; mechanochemical – red) and simulated (blue) PXRD traces of $[CdBr_2(4\text{-}pym)_2]_n$ (**5**)

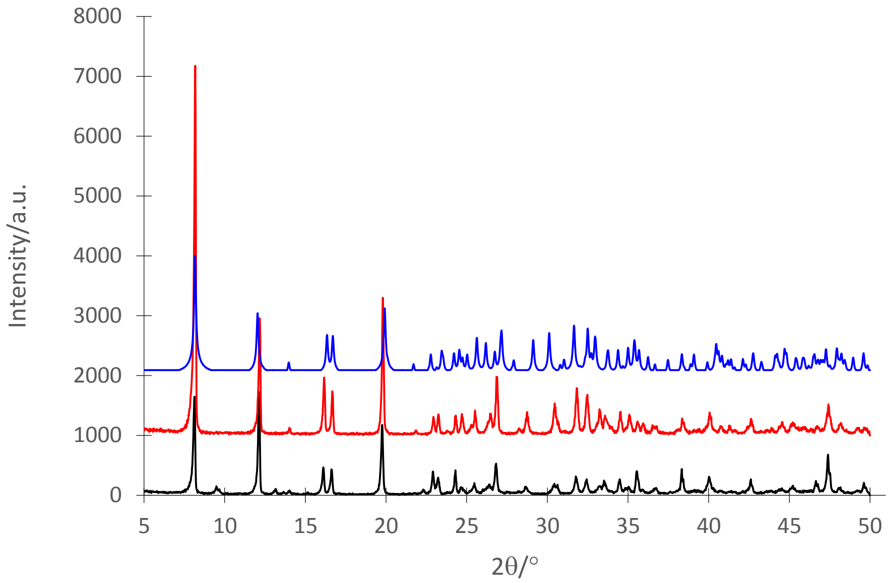

Figure S10 Experimental (solution – black; mechanochemical – red) and simulated (blue) PXRD traces of $[CdI_2(4\text{-pym})_2]_n$ (**6**)

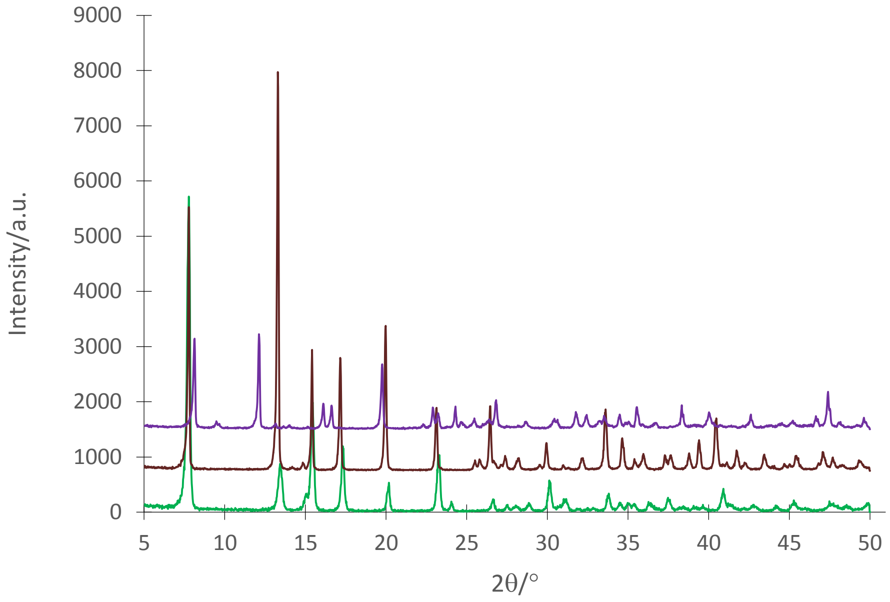

Figure S11 Overlay of experimental PXRD traces for $[CdCl_2(4\text{-pym})_2]_n$ (**4**) (green), $[CdBr_2(4\text{-pym})_2]_n$ (**5**) (brown) and $[CdI_2(4\text{-pym})_2]_n$ (**6**) (violet).

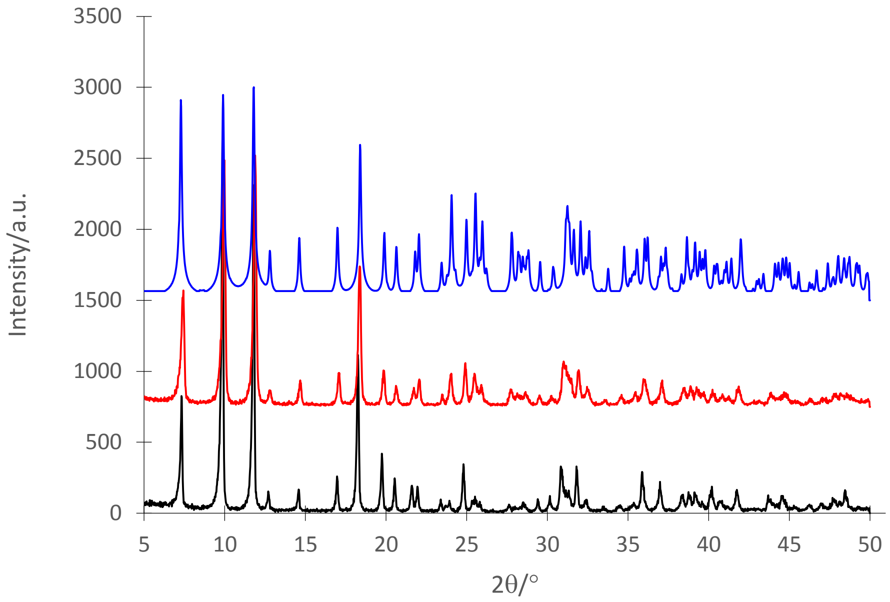

Figure S12 Experimental (solution – black; mechanochemical – red) and simulated (blue) PXRD traces of $[CdCl_2(4\text{-quz})_2]_n$ (**7**)

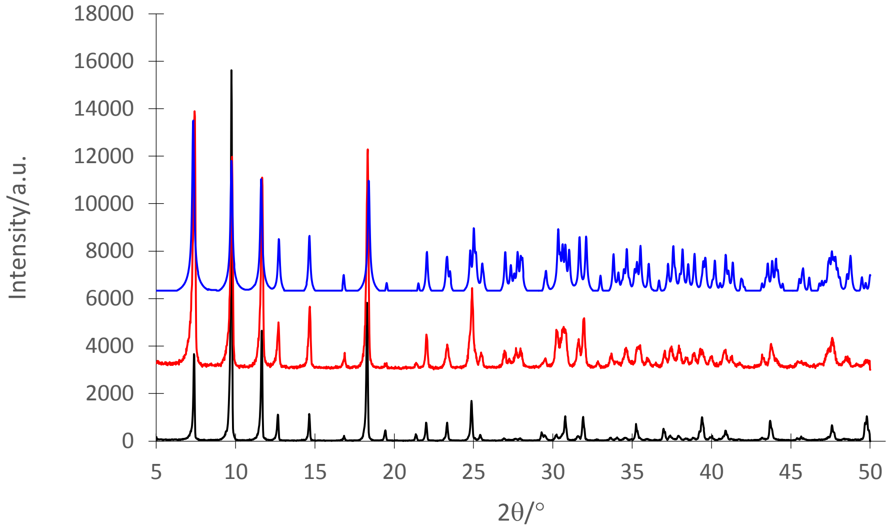

Figure S13 Experimental (solution – black; mechanochemical – red) and simulated (blue) PXRD traces of $[CdBr_2(4\text{-quz})_2]_n$ (**8**)

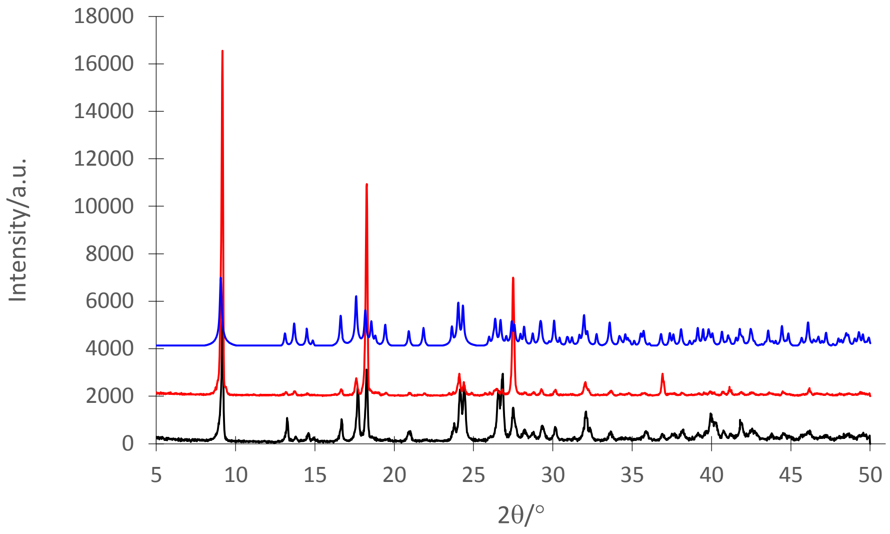

Figure S14 Experimental (solution – black; mechanochemical – red) and simulated (blue) PXRD traces of $[CdI_2(4\text{-quz})_2]_n$ (**9**)

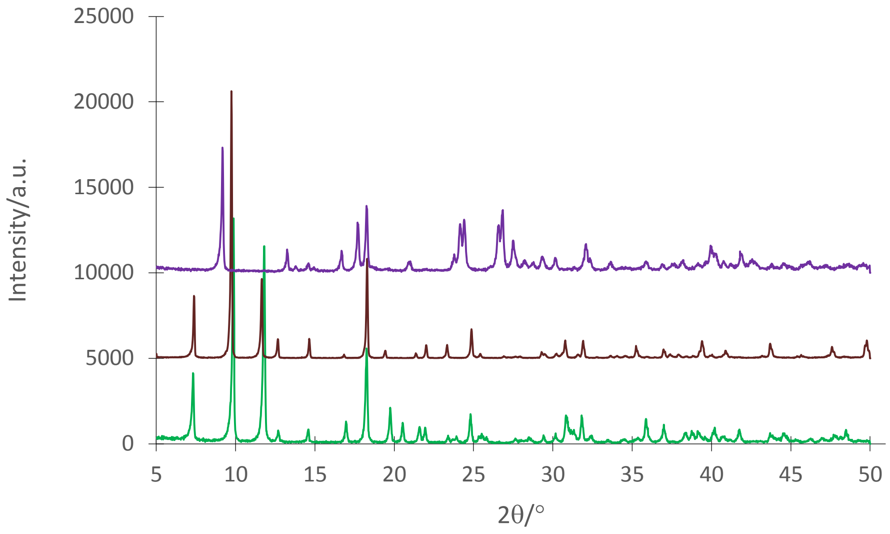

Figure S15 Overlay of experimental PXRD traces for $[CdCl_2(4\text{-quz})_2]_n$ (**7**) (green), $[CdBr_2(4\text{-quz})_2]_n$ (**8**) (brown) and $[CdI_2(4\text{-quz})_2]_n$ (**9**) (violet).

S4. QTAIM analysis

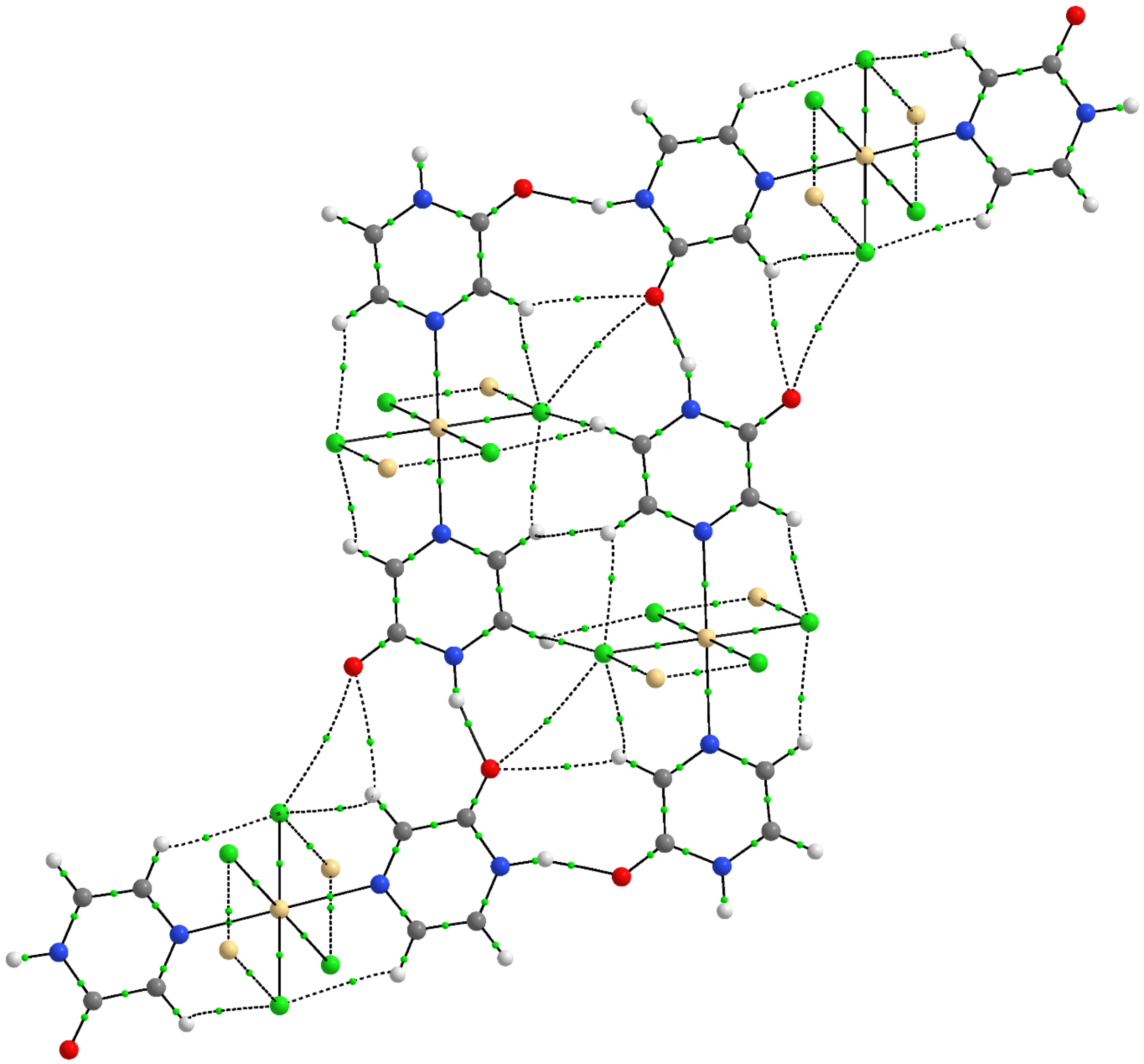

Figure S16 QTAIM analysis of **1**.

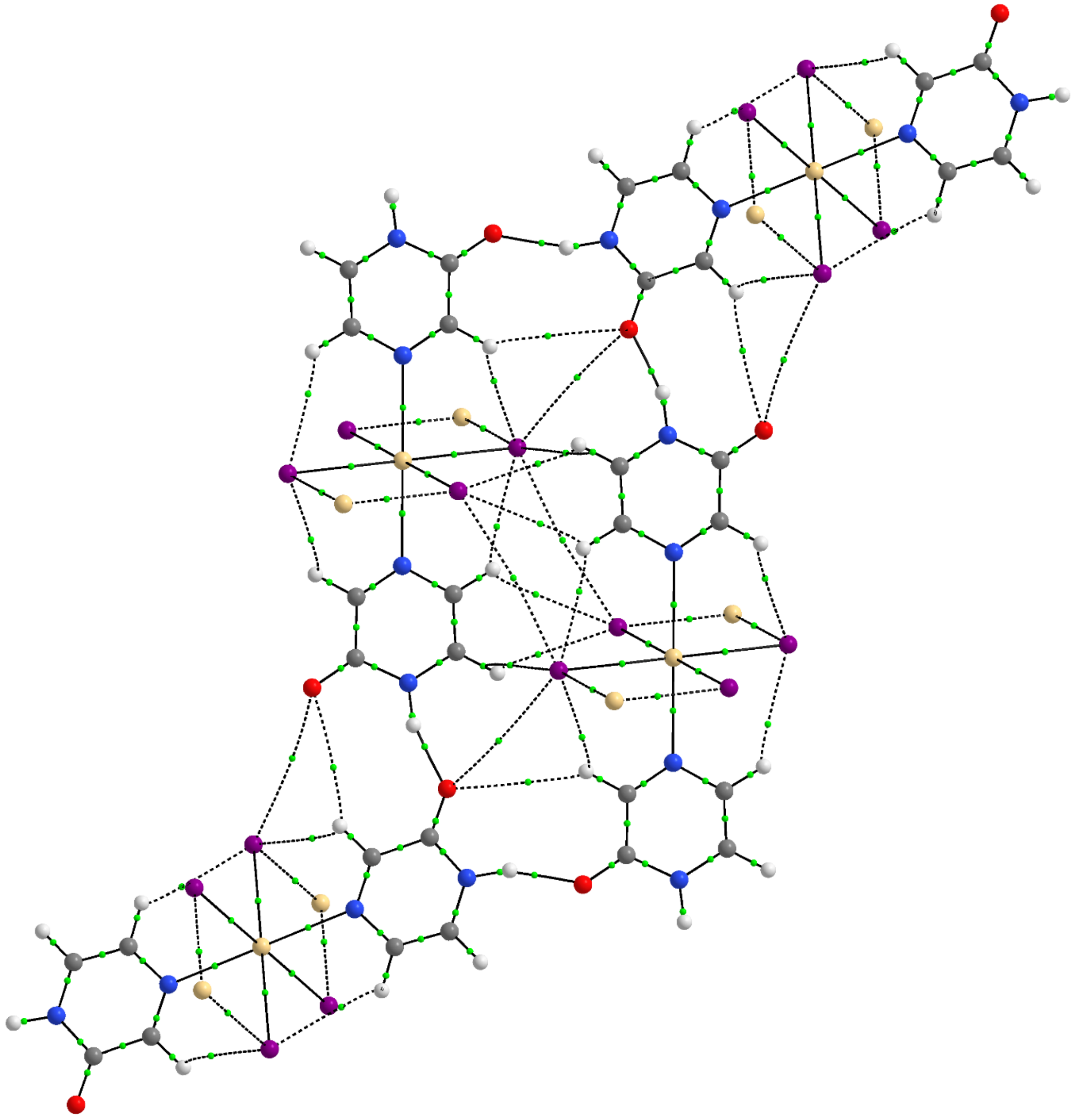

Figure S17 QTAIM analysis of **3**.

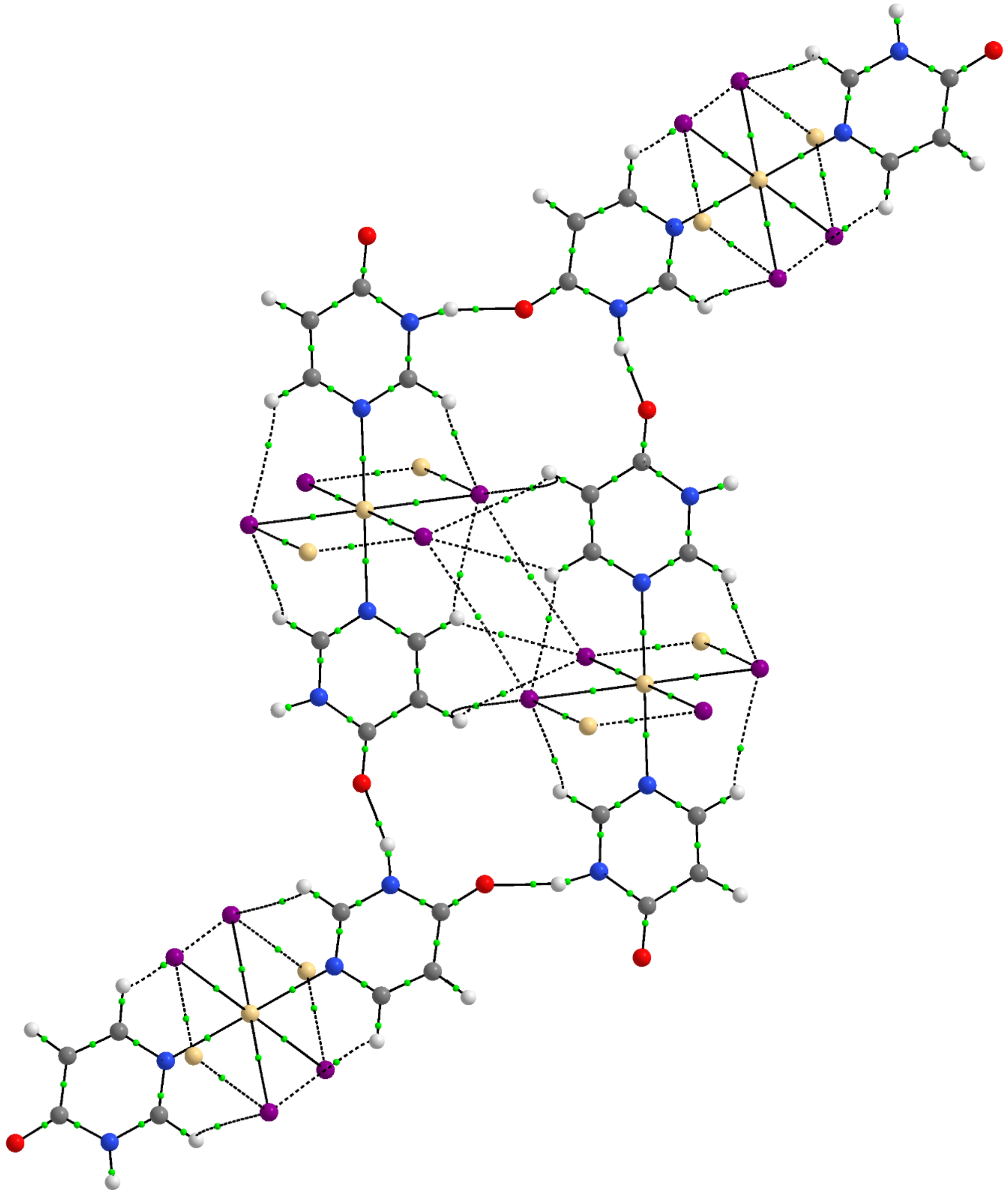

Figure S18 QTAIM analysis of **6**.

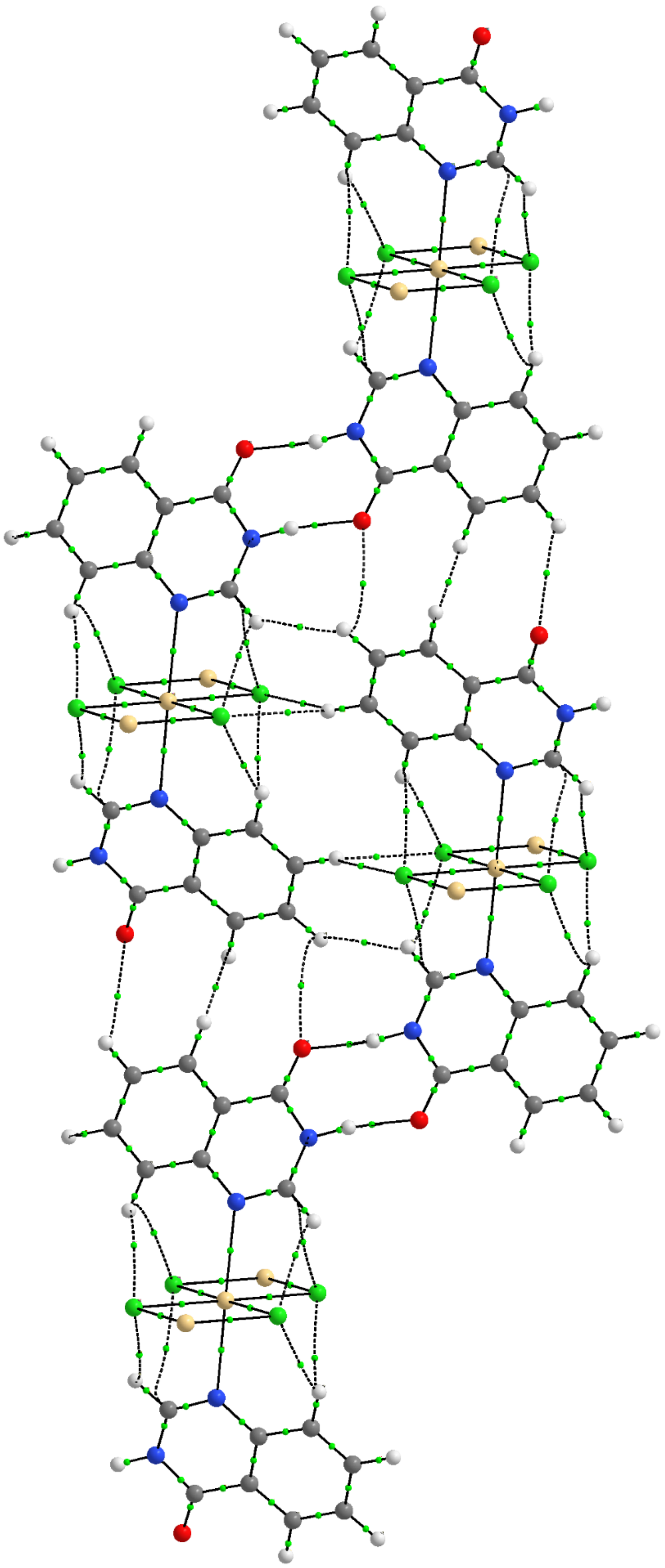

Figure S19 QTAIM analysis of **NALFEN**.
